# Genetic and Biotechnological Approaches to Improve Fruit Bioactive Content: A Focus on Eggplant and Tomato Anthocyanins

**DOI:** 10.3390/ijms25126811

**Published:** 2024-06-20

**Authors:** Maria Cammareri, Amy Frary, Anne Frary, Silvana Grandillo

**Affiliations:** 1 Institute of Biosciences and BioResources (IBBR), Research Division Portici, National Research Council of Italy (CNR), Via Università 133, 80055 Portici, Italy; maria.cammareri@ibbr.cnr.it; 2 Department of Biological Sciences, Mount Holyoke College, South Hadley, MA 01075, USA; afrary@mtholyoke.edu; 3 Department of Molecular Biology and Genetics, Izmir Institute of Technology, Izmir 35433, Turkey

**Keywords:** *Solanum melongena*, *Solanum lycopersicum*, wild species, genetic mapping, QTL, breeding, transgenic approaches, transcriptional regulation

## Abstract

Anthocyanins are a large group of water-soluble flavonoid pigments. These specialized metabolites are ubiquitous in the plant kingdom and play an essential role not only in plant reproduction and dispersal but also in responses to biotic and abiotic stresses. Anthocyanins are recognized as important health-promoting and chronic-disease-preventing components in the human diet. Therefore, interest in developing food crops with improved levels and compositions of these important nutraceuticals is growing. This review focuses on work conducted to elucidate the genetic control of the anthocyanin pathway and modulate anthocyanin content in eggplant (*Solanum melongena* L.) and tomato (*Solanum lycopersicum* L.), two solanaceous fruit vegetables of worldwide relevance. While anthocyanin levels in eggplant fruit have always been an important quality trait, anthocyanin-based, purple-fruited tomato cultivars are currently a novelty. As detailed in this review, this difference in the anthocyanin content of the cultivated germplasm has largely influenced genetic studies as well as breeding and transgenic approaches to improve the anthocyanin content/profile of these two important solanaceous crops. The information provided should be of help to researchers and breeders in devising strategies to address the increasing consumer demand for nutraceutical foods.

## 1. Introduction

**Figure 1:**
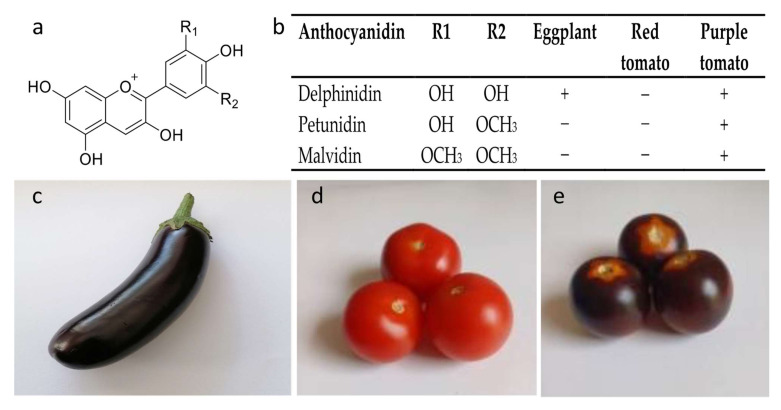
Basic anthocyanidin chemical structure (**a**) and the three most common anthocyanidins (**b**) found in eggplant fruit (**c**), in red tomato fruits (**d**), and in purple tomato fruits obtained through traditional breeding (**e**).

Anthocyanins are plant pigments belonging to the flavonoid family of polyphenolic compounds. More than 600 anthocyanins have been identified [1]. Each molecule consists of an anthocyanidin and one or more sugar residues, which may be acylated by aromatic or aliphatic acids. Additional modifications may include hydroxylation and methylation. Of the more than 20 anthocyanidins that have been identified, six (cyanidin, delphinidin, malvidin, pelargonidin, peonidin, and petunidin) are deemed common [2]. While cyanidin 3-glucoside is the most abundant anthocyanin in plants [3], delphinidin derivatives are the only naturally occurring anthocyanins in the two solanaceous species that are the focus of this review: eggplant (*Solanum melongena* L.) and tomato (*Solanum lycopersicum* L.) (Figure 1) (reviewed in [4]).

The absorption spectra of anthocyanins are amazingly variable, and their colors range from pinks and reds to shades of blue, violet, and black. This variability is due in part to the type, number, and position of the aforementioned chemical alterations [2]. In addition, as water-soluble molecules, anthocyanins are stored in the vacuole of plant cells, and the vacuolar pH impacts their color. More acidic conditions result in a shift toward red. Copigmentation, the noncovalent association of anthocyanins with organic molecules or metals, is another factor determining their absorption spectra and produces a shift toward blue.

The biological roles of anthocyanins in plants are nearly as varied as their chemical composition. Commonly produced in floral organs and fruit tissues, anthocyanins signal pollinators and dispersal agents. In vegetative tissues (leaves and stems), anthocyanin accumulation is often an indicator of stress and/or senescence. Cold temperatures, high light conditions, nutrient deficiency, and pathogen infection have all been shown to trigger anthocyanin synthesis [5,6]. The mechanisms by which anthocyanins help protect plants against biotic and abiotic stress are currently unclear. Their role in protecting against photoinhibition has been linked to their dual nature as pigments and antioxidants. As pigments, they can absorb excess light energy to help protect chloroplasts from photo-oxidative damage. As antioxidants, they scavenge reactive oxygen species that result from normal metabolism as well as biotic and abiotic stressors [6,7]. For example, it is likely that the antioxidant activity of anthocyanins provides benefits during episodes of biotic stress such as infection or herbivore attack [4].

The high antioxidant capacity of anthocyanins is similarly hypothesized to account for their purported roles in human health [1,3,5]. Studies have reported that anthocyanin extracts have anti-microbial, anti-inflammatory, and anti-tumorigenic effects [8]. In addition, benefits for diabetes [9], cardiovascular disease [10], gastrointestinal function [11], and ocular, brain [12], and liver health [13] have been reported. The value of anthocyanin extracts as natural colorants for food and cosmetics that could also improve the antioxidant activities of these products is being investigated [14,15].

**Figure 2:**
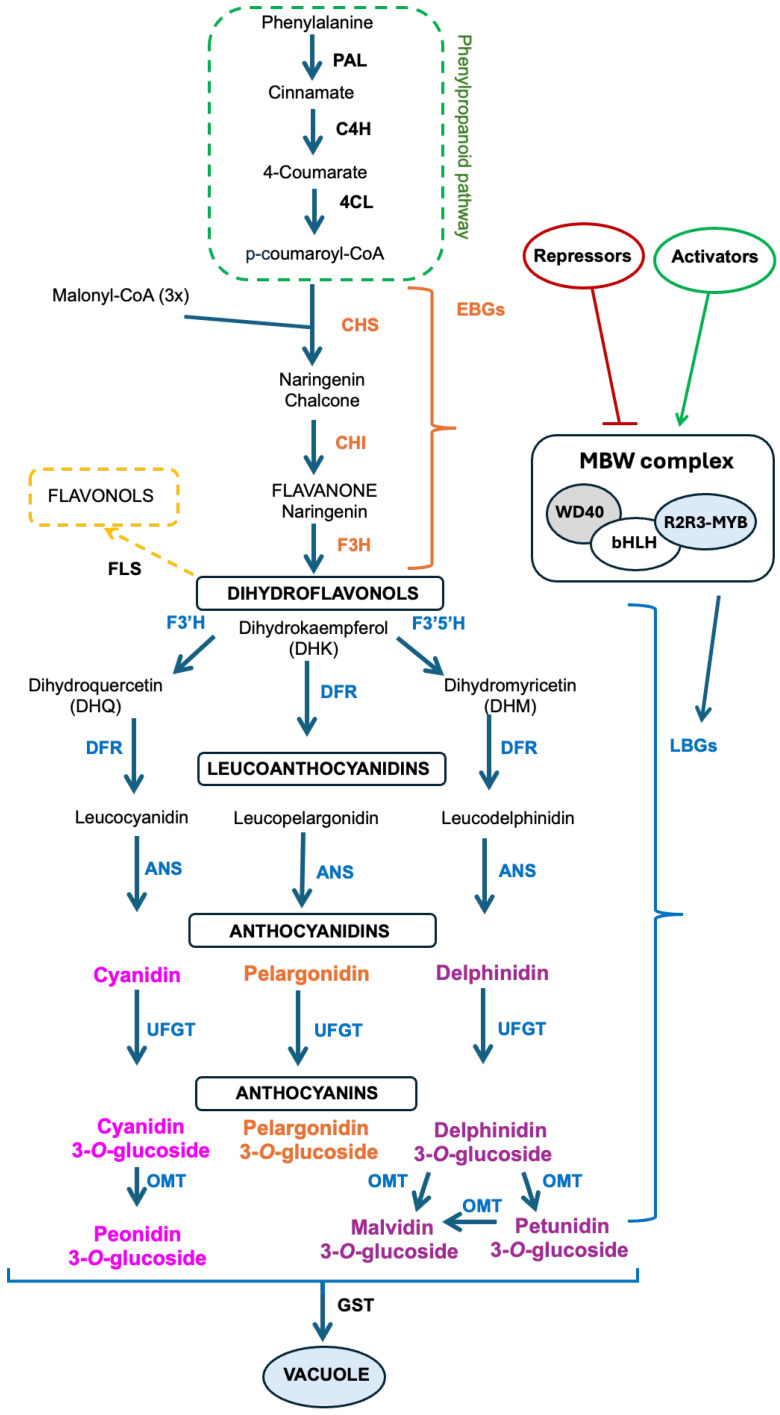
Schematic representation of the anthocyanin biosynthetic pathway. The phenylpropanoid pathway is the starting point of flavonoid biosynthesis through the catalytic activity of phenylalanine ammonialyase (PAL), cinnamate 4-hydroxylase (C4H), and 4 coumarate CoA ligase (4CL). The first step of the flavonoid pathway is the synthesis of the pale yellow-colored naringenin chalcone by chalcone synthase (CHS) using p-coumaroyl-CoA and three molecules of malonyl-CoA as substrates. Chalcone isomerase (CHI) then isomerizes chalcone into naringenin, a flavanone, which is a branching point for the formation of several groups of flavonoids. Conversion of naringenin into the flavonol dihydrokaempferol (DHK) is catalyzed by flavanone 3-hydroxylase (F3H). Different hydroxylases catalyze the production of varied dihydroflavonols from DHK; specifically, flavonoid 3′-hydroxylase (F3′H) and flavonoid 3′,5′-hydroxylase (F3′5′H) catalyze the production of dihydroquercetin and dihydromyricetin, respectively. Dihydroflavonols are converted into leucoanthocyanidins by dihydroflavonol 4-reductase (DFR), which has strict substrate specificity. Anthocyanidin synthases (ANS) transform these colorless compounds into the correspondent anthocyanidins as cyanidin (reddish-purple color), pelargonidin (orange), and delphinidin (purple). Finally, flavonoid 3-O-glucosyltransferase (UFGT) adds a sugar molecule to produce the anthocyanins (e.g., delphinidin 3-glucoside). The methylation of delphinidin by anthocyanin O-methyltransferases (OMT) leads to the biosynthesis of petunidin and malvidin derivatives, while the methylation of cyanidin leads to the synthesis of peonidin. Once anthocyanins are synthesized, they associate with glutathione-S-transferase (GST) for transfer into the vacuole via tonoplast transporters [21]. The molecular mechanism of entry into the vacuole is still being elucidated. Abbreviations: early biosynthetic genes (EBGs); late biosynthetic genes (LBGs).

Anthocyanin levels in plant tissues are the result of both biosynthesis and degradation and are regulated by several factors, including genotype, developmental stage, and environment [4]. The biosynthetic pathway of anthocyanins is well characterized and conserved throughout plants. The solanaceous species petunia (*Petunia hybrida* E. Vilm.) served as the model for elucidating the pathway [16,17]. Anthocyanins are synthesized in the cell cytoplasm via the flavonoid branch of phenylpropanoid metabolism, which starts with the amino acid phenylalanine (Figure 2). While more than 20 anthocyanidins have been detected in solanaceous species, mainly delphinidin-based compounds are found in eggplant and tomatoes [4,18,19].

Anthocyanin biosynthesis is mainly controlled by structural genes encoding pathway enzymes, regulatory genes encoding transcription factors (TFs), and the response of these genes to environmental signals. In general, light and stresses such as nutrient deficiency and low temperatures cause increased anthocyanin accumulation [20]. As anthocyanin regulation has been treated in several recent reviews (for example, [4,21,22,23]), this topic will be summarized here, and more specific information will be provided for eggplant and tomato in the following sections:

In dicotyledons, the early biosynthetic genes (EBGs) of the anthocyanin pathway (*CHS*, *CHI*, and *F3H*) are regulated by MYB TFs. The late biosynthetic genes (*F3′H*, *F3′5′H*, *DFR*, *ANS*, and *UFGT*) are regulated by the MYB–bHLH–WDR (MBW)–TF complex (Figure 2) [4,23]. The MBW–TF complex contains three types of TFs: MYB, basic helix–loop–helix (bHLH), and WD40. This complex is necessary to activate pathway genes [24]. In addition to activation, MYB TFs and the MBW complex can also repress anthocyanin biosynthesis [23]. The expression of these TFs is affected by environmental signals such as light, temperature, and nutrient availability, as well as endogenous factors such as tissue and developmental stage, and all of these effects are mediated by the relevant receptors and other proteins [4,22]. For example, increases in anthocyanin production due to high light intensity are mediated by red/far red, blue, and ultraviolet light receptors, which, in turn, suppress COP1, an E3 ligase that targets specific MYB TFs for degradation. In light, these TFs are not degraded and are able to activate anthocyanin production [21].

In addition to structural and regulatory genes, anthocyanin biosynthesis is also regulated by epigenetic factors, including microRNAs, long noncoding RNAs, DNA methylation, and histone modification [21,22]. Indeed, repression of anthocyanin biosynthesis appears to be much more complex than activation, with more than 12 repressor ‘modules’ described in the review of LaFountain and Yuan [22]. Anthocyanin levels are also controlled by degradation due to instability as well as enzymatic degradation. Factors that affect stability include the amount of B-ring hydroxylation, the location and extent of glycosylation and acylation, and the formation of complexes with iron and magnesium [4]. Enzymes involved in anthocyanin degradation may include laccase, polyphenol oxidase, class III peroxidases, and β-glucosidases [20].

This review focuses on work conducted to elucidate the genetic control of anthocyanin content and to modify anthocyanin levels in two *Solanum* species, eggplant and tomato. Interest in anthocyanins as nutraceuticals is providing breeders of these important solanaceous crops with additional motivation to enhance fruit anthocyanin production in their programs to address the growing consumer demand for nutraceutical foods. While purple-fruited cultivars are currently a novelty in tomato, anthocyanin levels in eggplant fruit have always been an important quality trait. As detailed in this review, this difference has influenced the genetic and molecular studies, as well as the breeding and transgenic strategies applied to improve the anthocyanin contents and profiles of these two important solanaceous crops.

## 2. Eggplant

### 2.1. Importance of Eggplant As a Crop

Eggplant is the general name for a crop that encompasses several solanaceous species, including *Solanum melongena* L., *Solanum aethiopicum* L., and *Solanum macrocarpon* L. [25]. *S. melongena* is the most commonly cultivated eggplant, while the other two species are primarily grown in Africa [26]. *Solanum melongena* has variably shaped fruit, which ranges from nearly spherical to snake-like, and a fruit color that varies from white to lavender to nearly black, depending on the concentration of anthocyanins in the fruit peel at commercial maturity. *Solanum aethiopicum* is known as scarlet eggplant due to its high carotenoid content [27], while *S. macrocarpon* has edible leaves and fruits that are green or white with a purplish tinge [28]. Because only *S*. *melongena* has high amounts of anthocyanins in its edible portion, the remainder of this review will discuss this species of eggplant.

*Solanum melongena* is grown worldwide, with over 59 million tons produced in 2022 [29]. It is important to note that most of this production is in highly populated countries like China and India and that nearly 25% of the crop is harvested in low-income countries with food deficits [29]. Thus, eggplant is a significant source of calories in the developing world and is valued for its culinary versatility as well as its nutritional value [30]. In addition to anthocyanins, eggplant is rich in other bioactive compounds, such as steroidal glycoalkaloids and phenolic acids, which have been used in the pharmaceutical and cosmetic industries [30]. Given the number of studies conducted on the anthocyanin contents of this crop, we have limited our scope to the last ten years of work.

### 2.2. Anthocyanin Content

In the past ten years, research on the anthocyanin content of eggplant peel has examined the effects of genotype, developmental stage, grafting, light, and season. A comparison of Turkish and Indian genotypes with different peel colors at commercial maturity revealed that those with black fruit had the highest levels of anthocyanins, followed in decreasing order by purple, green with purple stripes, and green and white-fruited varieties [31,32,33,34]. In terms of a specific anthocyanin profile, eggplant is often classified into two types based on the predominant compound in their skin, with the Japanese type rich in nasunin [delphinidin 3-(p-coumaroylrutinoside)-5-glucoside] and the non-Japanese type rich in delphinidin 3-rutinoside (D3R) [18]. For example, D3R was the major anthocyanin in the widely grown Turkish eggplant cultivars ‘Aydin Sıyahı’, ‘Kadife Kemer’, ‘Trabzon Kemer’ as well as the germplasm line CGN23829 [31,35]. In contrast, nasunin was the predominant anthocyanin in the cultivars ‘Birgah’, ‘Black Bell’, and ‘Black Moon’ [36]. In general, D3R types are reported to have higher anthocyanin content and darker purple fruit peel than nasunin types [37,38]. For example, Menella et al. [37] compared three D3R types with seven nasunin types and found that the D3R genotypes had approximately 3.5-fold more anthocyanin content.

Despite the categorization of genotypes as Japanese and non-Japanese types, recent research has revealed that the anthocyanin profiles of some genotypes are more complex. Wang et al. [35] found that line 14-345 was rich in both nasunin and delphinidin-3-glucoside (D3G). Metabolomic analysis of five eggplant varieties ranging in color from white to green to purple to black identified 32 different anthocyanins [34]. While D3R and nasunin were the most abundant compounds in black and purple-fruited varieties, multiple types of delphinidin glycosides and cyanidin glycosides, as well as a few petunidin, malvidin, and peonidin glycosides and one pelargonidin glycoside, were detected in the different genotypes. In another work, Zhang et al. [39] examined the peel of the purple Chinese cultivar ‘Zi Chang’ and found that it contained neither nasunin nor D3R. Instead, this cultivar contained two newly detected derivatives. The major anthocyanin was delphinidin-3-glucoside-5-dirhamnoside, while delphinidin-3-glucoside-5-(coumaryl) dirhamnoside was present at much lower levels. The presence of a wider range of anthocyanins and the importance of the anthocyanin/flavonoid ratio were emphasized by Yang et al. [40], who examined six cultivars with peel colors ranging from green and white to orange, lavender, and black–purple. As expected, the purple types had a higher total anthocyanin content at commercial maturity, with 14 different anthocyanins identified, including cyanidins and pelargonidins, as well as the expected delphinidins. Interestingly, non-purple types also contained a wide range of different anthocyanins, albeit at low concentrations. The authors hypothesized that the delphinidin/flavonoid ratio was the best indicator of peel color in their materials. It is clear that more genotypes must be evaluated in order to obtain a clearer understanding of the range of anthocyanin compounds that may be found in eggplant peel and their effects on color.

The anthocyanin profile also differs with the stage of fruit development. Eggplant is marketed at the commercially mature stage, which is not the same as physiological ripeness, a later stage when seeds are also mature. Unripe and commercially mature fruit had higher levels of anthocyanin compared to those that were physiologically ripe [35,36,37,38]. D3R lines showed greater reductions in anthocyanin content with physiological ripening, with total anthocyanin levels reduced by as much as 95% at the overripe stage [36,37,38]. A comparison of CGN23829 (D3R type) with line 14-345 (nasunin and D3G) indicated that total anthocyanins were only greater in CGN23829 at the commercially mature stage, despite the fact that the physiologically ripe fruit of this line appeared more purple than the brown fruit of 14-345 [35]. While nasunin content did not change significantly over these stages, both D3R and D3G significantly decreased during ripening in both lines. The brown color of 14-345 at the physiologically ripe stage was attributed to naringenin chalcone, a flavonoid that is upstream of the anthocyanins.

The effect of grafting on peel anthocyanin content has been studied using both wild eggplant and tomato rootstocks. Moncada et al. [41] used a *Solanum torvum* Sw. rootstock and four cultivars to determine the effect of grafting on eggplant quality. While the different cultivars used in the experiments did not significantly vary for total anthocyanin content before or after grafting, grafting resulted in qualitatively darker fruit. In contrast, anthocyanin content in the variety ‘Zheqie No. 10’ was reduced when grafted onto *S*. *torvum*, *Solanum sisymbriifolium* Lam., and *Solanum aculeatissimum* Jacq. rootstocks as compared to self-grafted plants [42]. Kacjan Maršić et al. [43] used a tomato rootstock with three eggplant cultivars and a landrace, resulting in a 33% reduction in total content during one of the two test years. This effect was attributed to the more vigorous growth of grafted plants, which caused the shading of fruits, thereby limiting anthocyanin production, which is stimulated by light.

Light is a known inducer of anthocyanin biosynthesis; however, some eggplant genotypes are non-photosensitive. Non-photosensitive types can accumulate pigment even in the dark. For example, a non-photosensitive line accumulated 83% as much anthocyanin when grown in the dark as compared to the light [44]. Dark treatment of photosensitive genotypes is useful to study the timing of anthocyanin synthesis. Li et al. [45] kept fruits bagged until maturity and found that pigment accumulation was apparent two days after light exposure, with the most rapid accumulation at day 5 and maximum content at day 12. The effect of season on anthocyanin content was examined by Kumari et al. [46], who found that pigment levels were higher in eggplants produced from August to February as compared to those produced during the summer. The increased levels were attributed to colder day and night temperatures, as it is well known that cold temperature stress results in increased anthocyanin synthesis.

Based on the aforementioned studies, it is clear that the chemical composition of anthocyanins in eggplant peel and flesh is much more complex than previously thought and that depth of color cannot entirely predict content. Thus, breeders who aim to alter this trait must be aware of the individual compounds present in their genotypes as well as their variability. Similarly, farmers must take into account the fluctuations in anthocyanin content due to developmental stage, grafting, shading, and climate conditions if they intend to market their fruit as rich in bioactive components. The public also must be aware of these differences if they are consuming eggplant as a functional food.

### 2.3. Genetic Mapping

Anthocyanin pigmentation has been a key trait in quantitative trait locus (QTL) studies since the very first molecular linkage maps were developed for eggplant [47,48]. Over the last ten years, a succession of QTL analyses performed with ever-increasing numbers of molecular markers have revealed that regions on two chromosomes are responsible for much of the variation in anthocyanin accumulation in eggplant tissues [49,50,51,52,53,54,55,56,57,58,59].

One of these regions, on chromosome 5 (E05), is associated with the presence of anthocyanins in vegetative tissues, such as hypocotyls, stems, leaf veins, and peduncles. In addition, this region affects the color of the corolla and fruit peel, including the color under the fruit calyx (an indicator of photosensitivity). Biochemical analyses have linked this same genomic region to levels of D3R and nasunin. A list of these QTLs and the associated references can be found in Table 1, with additional details in Table S1.

Chromosome 10 (E10) also carries loci for anthocyanin traits. These QTLs impacting anthocyanin traits appear to cluster in three locations on E10 (Table 2 and Table S1). The first of the regions on chromosome 10 (E10.1) is associated with the color of stems, leaves, leaf prickles, leaf veins, peduncles, calyxes, fruits, under calyxes, and peels next to the calyx. E10.2 is linked to the presence of anthocyanins in the stems, leaves, prickles, leaf veins, peduncles, and fruits. The most distal region, E10.3, contains QTLs for stem, hypocotyl, leaf, leaf vein, and corolla anthocyanins. Barchi et al. [60] found evidence of a selective sweep on chromosome 10 during domestication. They hypothesize that this sweep transformed eggplant from its white/green-fruited ancestral form to its purple cultivated form. This provides additional support for the importance of chromosome 10 loci in controlling eggplant fruit peel color.

The high-density, high-resolution molecular linkage maps recently developed for eggplant [54,57,58] will undoubtedly facilitate marker-assisted selection. And, while early analyses relied on synteny between the chromosomes of eggplant and tomato to associate QTLs with previously identified anthocyanin genes [49,51,52,53,61], whole genome assemblies and annotations improve the accuracy with which candidate genes underlying anthocyanin accumulation can be predicted. Thus, chromosome 5 QTL was correlated with a gene encoding UDP glucose anthocyanidin 5-0 glucosyltransferase (5GT) [49]. At least 18 additional candidate genes, representing nine functional categories, have been found in the genomic region corresponding to the E05 QTL cluster [54,56]. Some of these genes (coding for scopoletin glucosyltransferases and cytochrome P450) are known to be involved in phenylpropanoid synthesis. Additionally, an acetyl-CoA-benzylalcohol acetyltransferase (AAT) encoded in the region may convert D3R to nasunin, and a gene similar to *cryptochrome 1* may influence light-dependent anthocyanin synthesis [54,56]. The conversion of D3G to tulipan appears to be controlled by 3GT (RT anthocyanidin-3-O-glucoside rhamnosyltransferase), an enzyme mapped to E05 and linked to anthocyanin type (nasunin vs. D3G) [59]. Evidence suggests that a frameshift mutation in the gene for 3GT prevents the conversion of D3G to tulipan in the purple-fruited line PI 286106.

The recent availability of an annotated eggplant genome sequence has also allowed researchers to identify candidate genes on eggplant chromosome 10. An amino acid substitution in a WD repeat-containing protein seems to underlie an E10.1 QTL controlling fruit pericarp color [57]. WD proteins are critical components of the MBW transcription factor complex that regulates anthocyanin biosynthesis. Other E10.1 QTLs were discovered to lie in genomic regions containing two anthocyanin synthesis genes [*CHS* and *UDP glucose anthocyanidin 3-0 glucosyltransferase* (*3GT*)] as well as two MYB transcription factor genes (*AN2* and *ANT1*) [49].

More than 20 genes that are potentially linked to anthocyanin accumulation have been discovered in the E10.3 region [54,56,58]. These candidates include genes involved in pigment synthesis and regulation, such as five anthocyanidin synthase (ANS) candidates, an ankyrin repeat-containing protein, and two putative transcription factors (belonging to the MYB and BES1/BZR1 families) [54,58]. The E10.3 region also contains genes for proteins implicated in abiotic stress responses: a PYL4 abscisic acid receptor, protein phosphatase 2C, dehydration-responsive element-binding protein 2C, and phosphoinositide phosphatase [54,56]. Intriguingly, phosphoinositide phosphatases also play a role in moving materials into vacuoles. Finally, as many as five peroxidases within E10.3 could help modulate anthocyanin levels through degradation [54,56].

Genes on chromosome 10 also seem to control light-regulated anthocyanin synthesis. Mangino et al. identified a *COP1* gene in the 10.3 region [58]. He et al. [62] showed that a chromosome 10 locus controlling anthocyanin production under low light conditions co-segregated with a single nucleotide deletion. The candidate gene (*SmFTSH10*) codes for a protease with a putatively negative effect on anthocyanin synthesis. Luo et al. [63,64] also identified a chromosome 10 gene that apparently decouples anthocyanin synthesis from light exposure. The authors concluded that *EGP21875*, a homolog of *SmMYB113*, was most likely responsible for the less-photosensitive phenotype in both the *purple-in-the-dark* (*pind*) mutant and the cultivar ‘609’. While single nucleotide polymorphisms (SNPs) were not detected within the *EGP21875* gene sequence itself, the authors suggest that two intergenic substitutions (33 kb upstream and 13 kb downstream of the gene) may impact cis-regulation, activating this *smMYB113* homolog under dark conditions and turning on the anthocyanin biosynthesis pathway. Knowledge of the loci identified by He et al. [62] and Luo et al. [63,64] should make selecting for shade tolerance in peel pigmentation easier.

Recent results provide compelling evidence that two of the classic eggplant fruit pigmentation genes, *D* and *P*, originally described by Tatebe [65,66], have at long last been identified. You et al. [67] located fruit peel anthocyanin QTLs on chromosomes 8 and 10. The chromosome 8 QTL (corresponding to the classic gene *D*) was identified as the biosynthesis gene *SmANS*, whereas the chromosome 10 QTL (corresponding to *P*) harbored the regulatory gene *SmMYB1*. Each of the white-fruited parents was found to carry a different mutation in one of these two genes, accounting for the epistatic relationship observed in the F2 mapping population. Silencing of either gene reduced anthocyanin accumulation in the peel of F1 fruit, whereas overexpression led to pigmentation in calli derived from the parental lines. A missense mutation at position 65 in the first exon of *ANS* has been correlated with increased anthocyanin levels [68], highlighting that *ANS* is a likely target for the improvement of eggplant pigmentation.

While the majority of work to date has focused on chromosome 5 and 10 loci, a candidate gene for flavonoid 3′5′ hydroxylase, the enzyme that hydroxylates the anthocyanin precursor dihydrokaempferol, has recently been identified on E12 [59]. A defective version of this gene appears to prevent nasunin accumulation in the green-striped eggplant line WCGR112-8. Gaccione et al. [69] have recently published a comprehensive QTL map of eggplant that synthesizes work from 28 mapping and genome-wide association (GWA) studies. The reader is referred to that publication.

Together with the aforementioned mapping studies, this work helps breeders prioritize genotypes and genomic regions for anthocyanin manipulation using traditional methods by highlighting the most effective loci and their potential protein products.

### 2.4. Genetic Regulation of the Pathway

#### 2.4.1. Biosynthetic Pathway Genes

Quantitative real-time PCR (qRT-PCR) analyses of anthocyanin biosynthetic genes have revealed that expression of these genes (*CHS*, *CHI*, *F3H*, *F3′5′H*, *DFR*, *ANS*, *3GT*) is upregulated in darker purple versus lighter-colored eggplant fruit peel [32,34,39,40,45,70,71,72]. According to Zhou et al. [34], activation of the early biosynthetic genes plays a greater role in anthocyanin accumulation than the later genes. Other studies have determined the time course of light-induced anthocyanin synthesis. Sharma et al. [32] examined *ANS* activity in fruit from purple, green, and white genotypes. As expected, the purple-fruited line had the highest levels of *ANS* expression and greater increases in activity over the sampled time period (7, 14, and 21 days after anthesis) than the other colored lines. ANS activity was highest at 14 days after anthesis.

Light-induced anthocyanin synthesis is often studied by fruit bagging. The fruit of photosensitive varieties of eggplant remains white if they develop in the dark (i.e., are bagged). Shading is associated with the downregulation of the *CHS*, *CHI*, *DFR*, *ANS*, and *3GT* genes [73]. Exposure to light resulted in the rapid accumulation of anthocyanin in the fruit peel, with a peak in gene expression after only 4 h of light exposure [74]. A purple tint was visible within two days of unbagging, and peak anthocyanin levels were reached by 10 days [45]. RNA sequencing (RNA-Seq) and qRT-PCR analysis revealed a pattern in which anthocyanin pathway structural genes were initially upregulated (between days 0 and 5) and then downregulated (up to day 12) following exposure to light [45]. A comparison of these results with those from a non-photosensitive variety of eggplant showed that the synthetic genes are differentially expressed in the two varieties under dark and light conditions [49,73]. Thus, in the dark, *CHI*, *F3H*, *F3′5′H*, *DFR*, *ANS*, and *3GT* were transcribed at 200-fold higher levels in non-photosensitive eggplant fruit [49]. After light exposure, transcript levels remained unchanged or declined in the non-photosensitive type [49,74]. Clearly, anthocyanin synthesis is regulated differently in photosensitive and non-photosensitive varieties.

Analysis of EMS-induced and spontaneous eggplant mutants with white flowers and green fruits revealed that the anthocyanin-free phenotype was linked to two different SNPs within the *DFR* biosynthesis gene [75]. The SNP induced by mutagenesis caused alternative splicing and retention of the second intron in *DFR* transcripts. This finding was confirmed by an examination of splicing patterns in 145 variably pigmented eggplant accessions, which showed a correlation between improper splicing of *DFR* intron 2 and the incidence of white or green flowers and/or fruit. The second SNP was found to be naturally occurring in an association panel of 282 accessions [75]. This mutation was pinpointed to the MYB recognition site of the *DFR* promoter, which reduces *DFR* transcription and produces anthocyanin-free flowers and/or fruit. This study provides a useful example of how the analysis of artificially induced mutants and naturally occurring variants can shed light on the regulation of pigment production and how it has been modified by domestication and selection.

#### 2.4.2. Transcription Factor Genes

The transcription of anthocyanin structural genes requires at least one member from each of three different transcription factor gene families: *MYB*, *bHLH*, and *WD40*. Additional transcription factors associated with light-induced anthocyanin synthesis are also being elucidated.

##### MYB Transcription Factors

MYB TFs have received the most attention due to their great variety and multifaceted roles. The MYB TFs related to anthocyanin biosynthesis largely belong to two subfamilies: R2R3-MYB and R3-MYB [21]. As previously mentioned, MYB anthocyanin pathway activators are mainly R2R3-MYB TFs, while repressors can belong to either subfamily. The first MYB TF gene cloned in eggplant was *SmMYB*, an R2R3-MYB that was found to be expressed in purple fruit peel [76]. *SmMYB1* and *SmMYB2* were then cloned based on their similarity to tomato ANT1, a known R2R3-MYB regulator of anthocyanin synthesis [39,77]. It has now been established that the original *SmMYB* is the same as *SmMYB2* and that a 54 bp insertion in *SmAN2* is the only difference in these three genes [21]. Similarly, *SmMYB1* is the same as *SmeANT1* [21]. The SmMYB1 protein was found to interact with bHLH1 TFs from potato and eggplant in yeast two-hybrid assays [39,78,79], thus confirming its potential as a member of MBW complexes.

*SmMYB1* expression varies according to peel color, genotype, and tissue. *SmMYB1* was upregulated in the peel of purple cultivars as compared to white varieties [39,72]. Docimo et al. [78] reported that *SmMYB1* was expressed at low levels in all tissues of the purple Italian cultivar ‘Lunga Napoletana’, with the exception of stems that had high levels. However, according to transcript profiling of *SmMYB1* in line 67/3, the gene was most highly expressed in unripe fruit peel, with low expression in commercially mature peel and no expression in flowers [79]. The differences in *SmMYB1* expression in different genotypes may be attributable to allelic differences in the gene. For example, four SNPs were found to differentiate S*mMYB1* of ‘Lunga Napoletana’ from the same gene in ‘Zi Chang’, a Chinese purple cultivar [78]. The effect of *SmMYB1* on biosynthetic pathway genes has been assessed by transient or stable over-expression of *SmMYB1* in *Nicotiana benthiamana* leaves and regenerating eggplant shoots, respectively [39,78]. In both cases, the introduction of this TF gene caused increased expression of biosynthetic pathway genes (*CHS*, *DFR*, and *ANS*) and anthocyanin accumulation [39,78].

In contrast to *SmMYB1*, the expression of *SmMYB2* was not correlated with fruit color [39]. Instead, its expression was highest in flowers [78] and not present in unripe or commercially ripe fruits [79].

In their early work, Stommel and Dumm [70] examined the expression of genes they designated *MYB_B_* and *MYB_C_* based on their sequence similarity to tomato and potato *MYB* genes. To our knowledge, the correspondence between these genes and the other eggplant MYB TFs is unknown. *MYB_C_* levels were up to 288-fold higher in purple compared to white peel [70] and 95-fold higher in ‘Black Beauty’ compared to its green-fruited mutant [80]. Expression of this gene increased over fruit development, matching the increased expression of biosynthetic genes and the accumulation of anthocyanin [70]. In contrast, the expression level of *MYB_B_* was much lower than *MYB_C_* in purple fruit but still 10-fold greater than in white fruit. Moreover, *MYB_B_* transcript levels did not correlate with the expression of biosynthetic genes or anthocyanin accumulation.

Another R2R3-MYB, SmMYB113, is a positive regulator of anthocyanin biosynthesis and has been well-studied. In a photosensitive cultivar, its expression was upregulated in fruit peels within a few hours after light exposure [45,74]. Sequence analysis revealed light-responsive elements in the promoter of *SmMYB113,* and infiltration of this gene into tobacco leaves triggered anthocyanin accumulation [45]. Overexpression of *SmMYB113* in eggplant increased levels of anthocyanin in fruit peel and flesh [40,81]. Metabolic profiling of these anthocyanins revealed that they were mostly delphinidins, as expected in eggplant fruit. However, most of the delphinidins were glycosylated at the fifth position of the B-ring, an observation that agreed with the increased expression of specific glycosylation-modifying enzymes in the transgenic line. Transcriptomic profiling revealed a set of 276 genes that were differentially expressed in the *SmMYB113* overexpressing transgenic eggplants [81]. Anthocyanin structural genes were included among the upregulated genes. In addition, five of the differentially expressed genes (*SmCytb5*, *SmGST*, *SmMATE*, *SmASAT3*, and *SmF3′5′M*) were also associated with differences in peel color amongst a group of six eggplant cultivars. Functional analyses revealed that these genes contained MYB-binding elements in their promoters, could be transcriptionally activated by *SmMYB113*, and had a significant impact on anthocyanin synthesis in eggplant peel.

In addition to its involvement in light-responsive anthocyanin biosynthesis, *SmMYB113* interacts with C-repeat binding factors (CBFs), key regulators of the cold response in plants [82]. This interaction is associated with increased expression of the biosynthetic genes *CHS* and *DFR*. In a comprehensive analysis of MADS-box genes in eggplant, Chen et al. [83] identified six genes that are likely to be involved in anthocyanin biosynthesis due to their predicted MYB-binding sites and the correlation of their expression patterns with *SmMYB113* expression, as well as biosynthetic pathway and other transcription factor genes (*SmWRKY44*).

Various other MYB TFs have been identified by transcriptome profiling in eggplant. For example, Li et al. [74] detected 18 differentially expressed MYB TFs within a few hours after light exposure in photosensitive eggplant fruit. Positive regulators identified in this way or via similarity to Arabidopsis MYB TFs include *SmMYB32*, *SmMYB35*, *SmMYB75*, and *SmMYB86* [45,84]. *SmMYB75* is an R2R3-MYB with petal-specific expression [84]. Yeast one and two-hybrid analyses indicated that *SmMYB75* binds to the *SmCHS* gene’s promoter region and also interacts with SmTT8, a bHLH protein [84]. Overexpression of *SmMYB75* resulted in plants with increased anthocyanin content in all tissues, including eggplant flesh, and differential expression of several anthocyanin pathway genes [85].

SmMYB35 is another R2R3-MYB that can bind to the promoters of several eggplant anthocyanin genes: CHS, F3H, DFR, and ANS [21]. SmMYB94 and SmMYB19 can also bind directly or indirectly to the promoter of SmCHS [44]. SmMYB35 interacts with both SmTT8 and SmTTG1, a WD40 protein [86]. This interaction forms an MBW complex that triggers anthocyanin accumulation. Overexpression of SmMYB35 induced increased amounts of anthocyanin in petals and stems but not fruit. Most recently, ref. [87] characterized SmMYB5, a positive regulator of anthocyanin synthesis in response to jasmonic acid. This protein interacts with SmTT8 to activate the promoters of SmCHS, SmF3H, and SmANS. In contrast, interaction with SmCOP1 leads to its own degradation.

Compared to positive regulators, relatively few MYB TFs that repress anthocyanin synthesis have been identified. Transcriptomic analysis indicated that SmMYB44, SmMYB86, and SmMYB108 were downregulated by light exposure, suggesting their roles as repressors [45]. Further study of SmMYB86 indicated that the gene was expressed in leaves, stems, and peels and triggered by light [88]. Overexpression of this TF reduced anthocyanin accumulation, while silencing in the peel was associated with increased expression of CHS, F3H, and ANS and higher levels of anthocyanin. Yeast one-hybrid assays revealed that SmMYB86 binds to the promoters of these biosynthetic genes and suppresses their expression. It was also found to interact with the SmTTG1 protein and inhibit its binding to the promoter of the SmCHS gene. Moglia et al. [79] used sequence similarity to identify the repressor SmelMYBL1, an R3-MYB. This gene was expressed in flowers as well as unripe and commercially ripe fruit. When SmelMYBL1 was co-expressed with SmMYB1 and SmMYB2 in tobacco leaves, it repressed anthocyanin accumulation. The researchers hypothesized that SmelMYBL1 acts by competing with activator MYB proteins for bHLH binding sites, thus preventing the formation of activator MBW complexes. The binding of an R3-MYB in the place of an R2R3-MYB results in a repressor MBW complex.

Despite the wealth of our current knowledge, it is clear that much still remains to be discovered about eggplant MYB genes. Shi et al. [85] identified 102 R2R3-MYB genes in the genome and hypothesized that segmental duplication was the primary mechanism driving their evolution. In addition, miRNAs were identified that were predicted to target these TF genes. Expression of the R2R3-MYB genes under various abiotic stresses and hormone treatments was examined, demonstrating their diverse responses to such conditions.

##### bHLH Transcription Factors

The bHLH component of the MBW complex has received increasing amounts of attention. Like MYB proteins, bHLH TFs belong to a very large superfamily. Based on protein sequence similarity with Arabidopsis bHLHs, 121 eggplant candidate proteins were identified, including *SmbHLH1* and *SmbHLH117* [89]. In other work, transcript levels of one *bHLH* gene, *MYC*, were found to increase along with *MYB_B_* and *MYB_c_* levels during the various developmental stages of purple fruit but to be downregulated in a ‘Black Beauty’ mutant with green fruit [70,80]. *SmTT8* was found to have the highest expression in fruit peel [78], to be differentially expressed in purple vs. white cultivars [72], and to be upregulated in a photosensitive cultivar when exposed to light [45]. In contrast, both *SmTT8* and *SmMYC2* were highly expressed in the dark in a non-photosensitive cultivar with reduced expression in the light [44]. Yeast one-hybrid assays with both of these proteins indicated that they can bind directly or indirectly to the *SmCHS* promoter [44]. SmTT8 also interacts with SmMYB113 and increases anthocyanin accumulation in tobacco leaves when co-infiltrated with *SmMYB113* and *SmCBFs*, as compared to heterologous expression of *SmMYB113* and *SmCBFs* alone [82].

Expression profiles of *SmbHLH1* and *SmbHLH117* indicated that *SmbHLH1* was most highly expressed in purple tissues like peels and flower petals, while *SmbHLH117* showed the opposite pattern with the most expression in green tissues [89]. Analysis of the SmHLH1 amino acid sequence revealed that it only differs from SmTT8 at the N and C termini and is a negative regulator of anthocyanin accumulation, as indicated by overexpression experiments [90]. Moreover, SmHLH1 directly interacts with *SmDFR* and *SmANS* and does not interact with SmMYB113. Moglia et al. [79] examined the roles of two bHLH TFs in the MBW complex, *SmlAN1* and *SmlJAF13*. Both genes were highly expressed in unripe and commercially ripe fruit, with higher expression of *SmlJAF13* in flowers compared to *SmlAN1*. SmlAN1 was found to interact with the MYB TFs SmelAN2, SmelANT1, and SmelMYBL1. *SmbHLH13* was highly expressed in the leaves and flowers of eggplant and induced anthocyanin accumulation when overexpressed in Arabidopsis [91]. The protein was found to bind to and activate the promoters of both *CHS* and *F3H* in the anthocyanin pathway.

##### WD40 Transcription Factors

As previously mentioned, the WD40 component of the MBW complex does not directly interact with either MYB or bHLH proteins. Instead, it is believed to act as a platform for the interaction of other proteins [92]. A *WD40* gene named *WD* was found to be constitutively expressed in both purple and white-fruited cultivars but downregulated in a green-fruited ‘Black Beauty’ mutant [70,80]. *SmelAN11*, another *WD40* gene, was also found to be constitutively expressed in flowers as well as unripe and commercially ripe fruit [79].

##### Other Transcription Factors

In addition to MBW complex proteins, other TFs are involved in regulating anthocyanin synthesis. The WRKY family of TFs regulates many developmental, physiological, and stress response events and has a role in anthocyanin accumulation in Arabidopsis seeds [93]. In transcriptomics analyses, at least 15 *WRKY* genes, including *SmWRKY44* (also called *SmTTG2*), were differentially regulated in eggplant fruit after a photosensitive cultivar was exposed to light [45]. In non-photosensitive eggplant, both *SmWRKY44* and *SmWRKY53* were highly expressed before light exposure, and both proteins were capable of binding to the promoter of *SmCHS* [94]. Weighted gene co-expression network analysis indicated that *SmWRKY44* is a hub gene for the coordinated regulation of anthocyanin biosynthesis [94]. *SmWRKY44* expression was induced by light, and its levels correlated with anthocyanin levels in eggplant leaves, flowers, and peels. Overexpression of the gene in Arabidopsis resulted in anthocyanin accumulation, while transient expression in eggplant leaves also increased pigment levels. Interaction assays indicated that SmWRKY44 can interact with SmMYB1 and that it activates the promoters of *SmCHS*, *SmF3H,* and *SmANS*. Zhou et al. [34] found that *SmWRKY44* expression was positively correlated with *SmTT8*, *SmGL2,* and *SmGATA26* expression. While the exact functions of *SmGL2* and *SmGATA26* in pathway regulation remain unknown, silencing of these genes resulted in reduced anthocyanin accumulation.

The APETALA2/Ethylene Response Factor (AP2/ERF) superfamily of TFs is involved in many aspects of plant growth, development, and stress response and has been recently implicated in the regulation of anthocyanin biosynthesis. The expression patterns of a few *SmAP2*/*ERF* genes are positively correlated with anthocyanin accumulation, with upregulation occurring after light exposure [44,95]. Both of these studies indicated that these genes can interact with the *SmCHS* promoter region and activate its expression. More recently, mitogen-activated protein kinase 4 (MPK4) has been implicated in the light-responsiveness of anthocyanin biosynthesis in addition to its previously known roles in leaf development, stomatal movement, chlorophyll synthesis, and other aspects of plant metabolism [96]. Yeast two-hybrid experiments indicated that MPK4 interacts with MYB75 and thereby negatively regulates this TF. In addition, the knockout of SmMPK4 using editing via CRISPR-Cas9 resulted in decreased anthocyanin accumulation under intense light.

Because anthocyanin synthesis is often light-dependent, various photoreceptors are involved in its regulation. These include blue light receptor cryptochromes (CRY1, CRY2, CRY3) and UV receptors (UVR3, UVR8), whose genes are upregulated after light exposure in photosensitive eggplant fruit [44,45,71,74]. Long hypocotyl 5 (HY5) is a master regulator that acts downstream of these photoreceptors and can bind to the promoters of light-inducible genes [97]. This gene is upregulated by light and can bind the promoters of *SmCHS* and *SmDFR* [71,74]. Negative regulators of the light response include the central repressor COP1 and blue light inhibitors of cryptochromes (BICs).

In the dark, COP1 represses both HY5 and MYB TFs by being involved in their degradation [98]. In eggplant exposed to light, *SmCOP1* is downregulated [71]. Moreover, this protein has been shown to interact with SmCRY1, SmCRY2, SmHy5, and SmMYB1, thus confirming its importance in the regulation of light-dependent anthocyanin accumulation. RNAi of an additional COP1-interacting protein, SmCIP7, dramatically lowered levels of fruit peel anthocyanin [99]. This reduction was accompanied by the downregulation of anthocyanin structural genes and the regulatory gene *SmTT8*. Li et al. [99], therefore, hypothesized that *SmCIP7* acts as a positive regulator of light-induced anthocyanin synthesis via TT8.

BICs act by binding to CRY2, thereby inhibiting CRY2 dimerization when exposed to blue light [100]. *SmBIC1* and *SmBIC2* were upregulated in photosensitive eggplant fruit after light exposure [44]. Interaction analyses showed that SmBICs can bind to the promoter of *SmCHS* [94]. Moreover, silencing of these genes resulted in increased anthocyanin accumulation, while overexpression of either gene reduced accumulation. These alterations in anthocyanin levels were accompanied by the expected changes in the expression of anthocyanin biosynthesis structural genes and TFs, including *SmMYB1*, *SmTT8,* and *SmHY5*. This study also showed that the SmBICs interacted with SmCRY2 as expected based on previous research in Arabidopsis.

Clearly, the regulation of anthocyanin synthesis in eggplant is complex, and much remains to be elucidated. MYB-family transcription factors play a pivotal role in activating the anthocyanin biosynthetic pathway during fruit development (e.g., *MYB1*) and in response to environmental stressors (e.g., *SmMYB113*). Thus, these genes are the most logical targets for breeding programs aimed at enhancing the color of purple varieties and ensuring adequate pigmentation of fruit that develops under low-light/shaded conditions.

### 2.5. Transgenic Approaches

As explained in previous sections, genetic modification studies such as gene overexpression and silencing have helped to elucidate the roles of several structural and regulatory genes in the anthocyanin biosynthetic pathway. They have also revealed several ancillary effects of altering anthocyanin levels.

Transformation of *Solanum aethiopicum* gr. *gilo* (African or scarlet eggplant, which produces anthocyanin-free fruit) with the *SmMYB1* gene resulted in transgenic plants in which the late structural genes of the synthetic pathway were upregulated and vegetative, floral, and fruit tissues accumulated seven different anthocyanins, mostly delphinidin derivatives [101]. Interestingly, these plants also exhibited decreased water loss from detached leaves and increased tolerance to freezing stress (8 h at −5 °C), providing support to the hypothesis that the accumulation of anthocyanins in response to abiotic stress is adaptive. Overexpression of *SmMYB113* in a white cultivar yielded plants that accumulated anthocyanins in all tissues of the shoot system; additionally, 27 different anthocyanins (predominantly delphinidins) were detected in the fruit and peel of transgenic fruit [40].

Virus-induced gene silencing of the eggplant chalcone synthase (*SmCHS*) gene (performed by infiltrating the fruit stalks of a purple cultivar) resulted in fruit with white sectors [102]. The late structural genes *F3H*, *F3′5′H*, and *DFR* were also downregulated. Some unusual effects were observed in the *SmCHS*-silenced fruit: they were shorter and less straight than the wild type (WT), having a pronounced upward curvature. These effects were attributed to alterations in epidermal cell expansion and gravitropism, suggesting that *SmCHS* inhibition may have roles in both developmental phenomena. Perhaps these unforeseen effects are not surprising given the central role of chalcone synthase in the synthesis of flavonoids, a group of secondary metabolites with a multiplicity of roles in plant physiology, development, and defense. Therefore, it may be advisable to target late biosynthetic genes or genes involved specifically in delphinidin decoration for genetic modification, depending on the goal. For example, overexpression of the anthocyanin acyltransferase (*SmAAT*) gene was found to shift anthocyanin content from D3R (black–purple pigmentation) to nasunin (lilac pigmentation) [103].

The establishment of CRISPR-Cas9 technology in solanaceous species [104] means that it is now possible to edit key anthocyanin biosynthetic and regulatory genes. However, this work has been extremely limited in eggplant to date, with only one study in which targeted editing was used to help confirm the role of *SmMPK4.1* (a mitogen-activated protein kinase gene) in light-induced pigmentation [96]. We anticipate that an increasing number of studies will use this powerful technique to understand the genetic basis of anthocyanin synthesis. Gene editing also provides advantages over breeding methods to improve this key fruit quality trait, given the precision and specificity of the technique and the complex genetic control of the trait. Editing is also expected to be more acceptable to consumers than transgenic approaches, as it is evolutionarily more similar to the way that mutations occur in nature than the introduction of ‘foreign’ DNA.

## 3. Tomato

### 3.1. Importance of Tomato as a Crop

The cultivated tomato *Solanum lycopersicum* L., along with 12 wild relatives, belongs to the plant group *Solanum* sect. *Lycopersicon*, the tomato clade in the family Solanaceae [105,106]. Of these 13 species, only two (*S. lycopersicum* and *Solanum pimpinellifolium* L.) bear red fruit. Two other species (*Solanum galapagense* S. C. Darwin & Peralta and *Solanum cheesmaniae* (L. Riley) Fosberg) produce yellow to orange fruits, while the remaining species (*Solanum arcanum* Peralta, *Solanum chilense* (Dunal) Reiche, *Solanum corneliomulleri* J.F. Macbr., *Solanum pennellii* Correll, *Solanum peruvianum* L., *Solanum huaylasense* Peralta, *Solanum chmielewskii (*C.M. Rick, Kesicki, Fobes & M. Holle) D.M. Spooner, G.J. Anderson & R.K. Jansen), *Solanum habrochaites* S. Knapp & D.M. Spooner and *Solanum neorickii* D.M. Spooner, G.J. Anderson & R.K. Jansen) have green mature fruits. 

Tomato is one of the most economically important and extensively grown vegetable crops worldwide, with a cultivation area of ~5.0 million ha, yielding over 186.0 million tons per year in 2022 [107]. The top six tomato producers are China, India, Turkey, the USA, Egypt, and Italy, representing about 67% of the world production [107]. 

Several factors have contributed to the worldwide cultivation of tomatoes, including the species’ adaptability to a variety of climatic and growth conditions (both in open fields and greenhouses) and the product’s versatility (it can be consumed both fresh and as processed products like soups, juices, and sauces) [108]. In recent decades, there has been a significant increase in tomato consumption, promoted by its numerous bioactive compounds, for which this fruit was identified as a ‘functional food’ [109]. 

### 3.2. Anthocyanin Content in Cultivated Tomato

Tomato berries have a high nutritional and nutraceutical value, being a rich source of essential nutrients and phytochemicals, including minerals (mainly K, P, and Mg), soluble sugars, organic acids, dietary fibers, vitamins (particularly vitamin C and vitamin E), carotenoids (lycopene, β-carotene, and lutein), polyphenols (chlorogenic acid, quercetin, and naringenin), as well as volatile compounds [108,110,111,112]. The chemical composition and overall quality of tomato fruits are determined by numerous factors, including the genotype, environmental conditions, cultural practices, ripening stage, and post-harvest handling and processing operations [113,114].

The beneficial properties of tomatoes in preventing chronic degenerative disorders are largely associated with the chemo-preventive and anti-proliferative activities of their antioxidants [115]. Several metabolites contribute to the high antioxidant capacity of tomato fruits, including both lipophilic (carotenoids and vitamin E) and hydrophilic (vitamin C and phenols) compounds [116]. Foods rich in both hydrophilic and lipophilic antioxidants are generally thought to provide the best defense against diseases [117,118]. In tomatoes, the lipophilic carotenoids represent the main secondary metabolites, with the red-colored lycopene being the most abundant one. In contrast, the concentrations of water-soluble flavonoids are considered suboptimal, with only small amounts (~5–10 mg/kg fresh weight) of some flavonoid biosynthetic intermediates largely synthesized in the epidermis, while the fruit flesh, which represents 95% of total fruit weight, normally does not produce any flavonoids [19,119,120,121]. The main flavonoids found in tomato peel are the yellow-colored naringenin chalcone (the most abundant flavonoid in red tomatoes) and the flavonol glycosides rutin (quercetin rutinoside) and kaempferol-3-O-rutinoside, along with small amounts of a quercetin trisaccharide [115,120]. Notably, unlike the fruit of other Solanaceae, such as eggplant (*Solanum melongena* L.) or pepper (*Capsicum* spp.), cultivated tomato fruit does not usually contain anthocyanins [19,119,120,121], and the red color is mainly due to the higher accumulation of the carotenoid all-*trans*-lycopene [122].

Besides red cultivars, tomatoes are found in a wide range of colors, which result from a combination of different pigments accumulating in the epidermis, the sub-epidermal layer, and the fruit pericarp (flesh) [123,124]. Several pink, orange, yellow, brown, black, green, purple, and even white tomato heirloom or hybrid genotypes have been described [19,125,126,127]. However, the heirloom varieties of tomatoes described as “purple” and “black”, do not contain anthocyanins [19,124,125], and their purplish-brown fruit colors are instead attributed to interactions of the carotenoid-based pericarp color (regulated by multiple genes) with the presence or absence of the mutations *colorless fruit epidermis* (*y*), *uniform ripening* (*u*), and *green flesh* (*gf*) [19]. In particular, *green flesh* (*gf*) inhibits the normal degradation of chlorophyll, resulting in the accumulation of the brown pigment pheophytin, which, when combined with the red of lycopene, gives a “dirty purplish brown” [19,124,128,129].

On the other hand, the vegetative tissues of cultivated tomato genotypes generally produce a variety of flavonoids, including anthocyanins. These compounds are synthesized during development and in response to several environmental stimuli [19,112]. Ibrahim et al. [130] first characterized the main anthocyanins in tomato vegetative tissues: petunidin, malvidin, and delphinidin. Subsequently, Bovy et al. [131] identified three anthocyanins in the vegetative tissue of light-stressed tomato seedlings: petunidin 3-(p-coumaroyl rutinoside)-5-glucoside (petanin), malvadin 3-(p-coumaroyl rutinoside)-5-glucoside (negretein), and petundin 3-(caffeoyl) rutinoside-5-glucoside.

Given the economic significance of tomatoes, much effort has been made to expand the range of the fruit’s bioactive compounds and increase the nutraceutical value of this important crop. Considering the health-promoting aspect of flavonoids, and specifically of anthocyanins, and the low content or even absence of these compounds in cultivated tomato fruits, this species has become a system to study the regulatory network of anthocyanin biosynthesis. The ultimate goal of such research is the activation of the flavonoid pathway to obtain anthocyanin-enriched tomato fruit using both traditional breeding and transgenic approaches for genetic manipulation [132].

### 3.3. Genetic Resources with Altered Anthocyanin Content

While the fruits of domesticated tomato and its red-fruited wild progenitor, *S. pimpinellifolium*, generally do not contain anthocyanins in the fruit flesh or peel, purple pigmentation of the peel, attributed to the anthocyanins petunidin and malvidin, is a characteristic found throughout the green-fruited wild species of the tomato clade [77,125,133]. In addition, anthocyanin-pigmented fruits have been observed in *S. cheesmaniae* and *S. galapagense* accessions [134,135,136,137], as well as in the green-fruited *Solanum lycopersicoides* Dunal, a distantly related wild nightshade species belonging to the immediate outgroup *Solanum* sect. *Lycopersicoides* [138]. In particular, *S. chilense*, *S. lycopersicoides*, and *S. cheesmaniae* have been the donors of three allelic variants (*Anthocyanin fruit* (*Aft*), *Aubergine* (*Abg*), and *atroviolacium* (*atv*), respectively) able to cause intensification of anthocyanin pigmentation once introgressed into a cultivated tomato background (see Section 3.4) [19,139] (http://tgrc.ucdavis.edu (accessed on 6 June 2024)).

In addition, several other tomato mutations, spontaneous or induced by physical or chemical mutagens, are known to alter anthocyanin content/profile in the fruit or in vegetative tissues [139]. For instance*,* the mutations *anthocyanin less* (*a*), a*nthocyanin absent* (*aa*), *entirely anthocyanin-less* (*ae*), *anthocyanin free* (*af*), *anthocyanin gainer* (*ag*), *incomplete anthocyanin* (*ai*), *anthocyanin loser* (*al*), *anthocyanin reduced* (*are*), *without anthocyanin* (*aw*), and *baby lea syndrome* (*bls*) exert a negative effect on the production of anthocyanins in vegetative tissues ([139]; http://tgrc.ucdavis.edu (accessed on 6 June 2024)). Other tomato mutants display anthocyanin alteration as a secondary phenotype; this group includes the tomato photomorphogenic *high pigment* (*hp*) mutations (*hp-1*, *hp-1^w^*, *hp-2*, *hp-2^j^*, and *dg* or *hp-2^dg^*), which, besides exerting a positive effect on carotenoid contents in ripe-red fruits, are also known to influence anthocyanin levels in vegetative tissues [125,140,141].

The underlying genes for some of the abovementioned tomato mutants have been identified, including those for *anthocyanin absent* (*aa*) [142], *anthocyanin without* (*aw*) [143], *anthocyanin free* (*af*) [144], *anthocyanin reduced* (*are*) [145,146], and *Hoffman’s anthocyaninless* (*ah*) [147]*,* which encode a dihydroflavonol 4-reductase (DFR), a chalcone isomerase (CHI), a flavonoid 3-hydroxylase (F3H), and a bHLH TF [147], respectively [139,148]. Also, as will be described in Section 3.6.2, the molecular basis of *atv* [148,149], *Aft* [150,151,152], and *Abg* [153] have also been identified.

Besides a well-characterized mutant collection, tomato breeding and biotechnological efforts can take advantage of a wealth of tools and resources [154,155], including a reference genome sequence of cultivated tomato [156] and genome sequences of numerous cultivated accessions and several related wild species [157,158,159,160,161]. Furthermore, tomato is among the best-characterized crop species at the metabolomic level [162] and has emerged as a main model for studying flavonoids and phenylpropanoids [162,163]. These advantages, together with the tomato’s amenability to genetic transformation and genome editing [164,165,166], have made this important crop an ideal system for developing anthocyanin-enriched fruit using both traditional breeding and genetic engineering approaches [155].

### 3.4. Traditional Breeding Efforts

Over the past decades, classical breeding activities based on interspecific crosses with wild species have successfully led to the establishment of various tomato lines and cultivars with anthocyanin-enriched purple-skinned fruits (Table 3 and Table S2). The first efforts started in the 1960s and 1970s, when plant geneticists crossed cultivated tomatoes with some wild relatives to transfer alleles for fruit skin anthocyanin production [167,168,169].

The dominant *Anthocyanin fruit* allele (*Aft*, formerly *Af*) was introgressed into cultivated tomato from the related wild species *S. chilense* [125,168]. The derived mutant *Aft*/*Aft* line (accession LA1996) [168], carrying *Aft* in an undeclared background, is one of the lines most frequently used to breed anthocyanin-based purple-peel fruits [19,170,171]. The *Aft* phenotype is regulated by a single dominant gene [125,168] located on the distal region of the long arm of chromosome 10 [19,172]. Anthocyanin-spotting in the fruit peel is produced upon exposure to intense light [170]. *Aft* plants exhibit some common phenotypic traits with other tomato photomorphogenic mutants (*hp-1*, *hp-2*, and *dg* or *hp-2^dg^*). However, unlike these high pigment mutants, the ripe fruits of LA1996 do not have a higher carotenoid content, and the unripe fruits are not dark green [125].

Anthocyanins produced in *Aft* LA1996 fruit were similar in composition to those from vegetative tissues and consisted mainly of petunidin, followed by malvidin and delphinidin (Table 3 and Table S2) [125]. *Aft* LA1996 fruits also had significantly higher levels of the flavonols quercetin and kaempferol, which increased their value as functional fruits [172].

Anthocyanin accumulation in the *Aft* LA1996 fruit epidermis was reported to be induced by chilling stress [168] and synergistically by blue and UV-B light [173]. Introgression of the dominant *Aft* allele into domesticated tomato induced a shift from flavonol to anthocyanin production in response to post-harvest UV-B treatment [174]. Recently, the anthocyanin-rich tomato genotype *Aft* LA1996 was shown to be more drought- and salinity-tolerant [175].

A second dominant allele causing anthocyanin production in fruit, *Aubergine* (*Abg*), was introgressed into cultivated tomato from *S. lycopersicoides* (accession LA2408) [138]. *Abg* was mapped to chromosome 10, on the same chromosome arm as *Aft* [138]. However, in crosses with cultivated tomatoes, *S. lycopersicoides* shows a paracentric inversion for this region of chromosome 10 [176,177], which has precluded the achievement of stable homozygotes of *Abg* in *S. lycopersicum* [125,177]. As a result, the genetic makeup of the *Abg* locus has long been unknown. *Abg* and *Aft* share a similar phenotype in that they both need strong light to cause the synthesis of anthocyanins; however, they show different patterns of anthocyanin expression in and beneath the epidermal cell layer [19,125]. Unlike *Aft*, *Abg* displays variable expression, which can range from a blotchy, spotted pattern to a fully homogeneous, intensely pigmented appearance at maturity, even in different fruits of the same plant [19,138]. Notably, among the tomato anthocyanin mutants, *Abg* can exhibit, under adequate light exposure, some of the strongest pigment expressions, which justifies the name *Aubergine*, an allusion to the purple color of eggplant’s fruit skin.

Additionally, a recessive gene, *atroviolacium* (*atv*), derived from *S. cheesmaniae*, was shown to enhance anthocyanin pigmentation throughout the plant, especially in the vegetative tissues, when introgressed into cultivated tomatoes [19,169]. Furthermore, *atv*/*atv* plants exhibited an exaggerated response to red light in terms of anthocyanin production and hypocotyl growth inhibition, indicating that the *atv* mutation may be involved in phytochrome responses [178]. The *atv* locus was mapped to chromosome 7 of tomato [169]. 

Although the *atv* mutation *per se* has no important effect on the accumulation of anthocyanins in the fruit, a considerable increase in anthocyanin pigmentation in the fruit peel was obtained by combining *atv* with either *Aft* or *Ab*g (Table 3 and Table S2). Small fruit of the genotypes *Abg*/*-atv*/*atv* and *Aft*/*Aft atv*/*atv* displayed the highest level of anthocyanin expression in the fruit peel, with contents up to 4.2 and 1.2 mg/g FW, respectively [19]. In addition, the anthocyanin content did not influence carotenoid profiles or quantities [19]. Based on phenotypic and transcript profiling analyses of a double mutant *Aft*/*Aft atv*/*atv* line, in comparison with the single mutants and wild-type genotype, Povero et al. [179] hypothesized a synergistic effect of the two alleles, *Aft* and *atv*.

The most stable anthocyanin-rich fruit genotypes, *Aft*/*Aft atv*/*atv* [19], were used as breeding material to obtain the popular purple tomato cultivar ‘Indigo Rose’, suitable for fresh consumption, which was released in 2011 by the Oregon State University vegetable breeder Jim Myers [171]. Since then, Myers has further improved his line and has introduced four more purple tomatoes: ‘Indigo Cherry Drops’, ‘Indigo Pear Drops’, ‘Indigo Kiwi’, and ‘Midnight Roma’ (https://ourimpact.oregonstate.edu/story/oregon-state-vegetable-breeder-goes-wow; accessed on 22 May 2024). Consistent with its genetic composition, ‘Indigo Rose’ only produces anthocyanins in the part of the peel that is exposed to sunlight, and no anthocyanin accumulates in shaded peels or in the flesh [180]. The total content of anthocyanins reached 4.0 mg/g FW in the ‘Indigo Rose’ fruit peel, with petanin and malvidin-3-(trans-p-coumaroyl)-rutinoside-5-glucoside (negretein) being the two main anthocyanins (Table 3 and Table S2) [181].

**Figure 3:**
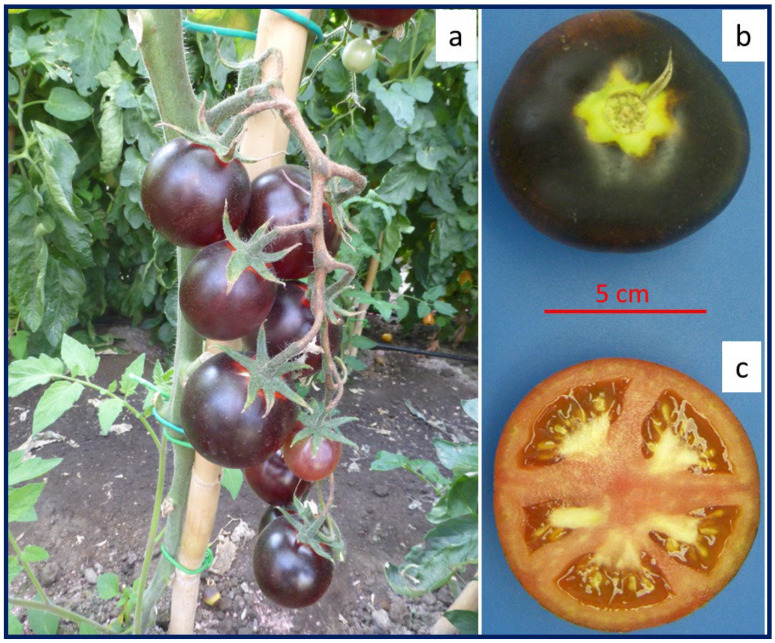
‘Sun Black’ (SB) tomato plant with a cluster of fruits (**a**), whole fruit (**b**), and transversal section (**c**) of SB fruit at the red ripe stage. Photographs provided by Andrea Mazzucato, Università della Tuscia, Viterbo, Italy.

Besides ‘Indigo Rose’ and its derivatives [133,182,183,184] (Table 3 and Table S2), the allelic combination *Aft*/*Aftatv*/*atv* was introduced into a different genetic background and led to the development of the ‘Sun Black’ selection, so-called for the light conditional deep, anthocyanin-based, purple pigmentation of the fruit peel (Figure 3) [170,179,185]. The name ‘Sun Black’ has been protected as a trademark, and the cultivars selected from the Tuscia University breeding program are now commercialized (cv. ‘Solenero’ and others) [186]. Besides high anthocyanin contents in the fruit peel (Table 3 and Table S2), the levels of other flavonoids increased in proportion to the anthocyanin concentration in ‘Sun Black’ fruits, resulting in a high hydrophilic antioxidant capacity, while the total carotenoid content was comparable to that of non-anthocyanin varieties [186].

Several studies have combined high-anthocyanin mutants (mainly *Aft* and *atv*) with those affecting carotenoid composition and content (e.g., *hp-1*, hp-2, *dg* or *hp-2^dg^*) to investigate the interactions between flavonoids and carotenoids and to obtain near-isogenic lines (NILs) with improved nutritional value (Table 3 and Table S2) [19,172,187,188,189]. A particularly promising combination was the triple mutant *Aft*/*Aft atv*/*atv hp-2*/*hp-2* NIL in ‘Micro-Tom’ (MT) background, resulting in uniformly dark purple fruit with increased levels of anthocyanins, *β*-carotene, lycopene, and vitamin C [187]. Once transferred from MT into a commercial, red-fruited cherry tomato cultivar, the three mutant alleles (*Aft*/*Aft atv*/*atv hp-2*/*hp-2*) produced purple fruits and enhanced nutrient contents [187,189]. In particular, the purple tomato peel accumulated high levels of anthocyanins, mainly petanin (Table 3 and Table S2), and other flavonoids such as rutin and kaempferol [189]. In addition, when compared to the red tomatoes, the peel and flesh of the purple tomatoes had considerably higher levels of lycopene and *β*-carotene, which the authors partly attributed to the increased number of chloroplasts (and thus chromoplasts) caused by the *hp-2* allele [187]. However, the presence of the three allelic variants for anthocyanin biosynthesis regulatory genes (*Aft*, *atv*, and *hp-2*) was also associated with appreciable alterations in volatile compound profiles. The effects of these changes on tomato flavor and consumer acceptability need to be assessed [189].

**Table 3:** Breeding achievements to induce/improve tomato fruit anthocyanin content. Allelic combination of the main anthocyanin-enriched tomato lines, total anthocyanin content. Abbreviations: FW, fresh weight; DW, dry weight. NA, not available.

Allelic Combination	Tomato Line (*S. lycopersicum* Genetic Background/Original Cross)	Total Anthocyanin Content	Reference
*Aft*/*Aft*	LA1996 (undeclared background)	0.66 mg/g FW (skin); 0.2 mg/g FW (pigment-rich pericarp beneath the skin)	[125]
*Aft*/*Aft*	LA1996 (undeclared background)	0.72 mg/g FW (skin) (2004); 0.18–0.36 mg/g FW (skin) (2006)	[19]
*Aft*/*Aft atv*/*atv*	(*Aft*, LA1996 × *atv*, LA0797)	1.2 mg/g FW (skin)	[19]
*Abg- atv*/*atv*	(*Abg* LA3668 × *atv*, LA0797)	4.2 mg/g FW (skin)	[19]
*Aft*/*Aft atv*/*atv*	‘Indigo Rose’	4.0 mg/g FW (peel extract); 0.09 mg/g FW (flesh extract)	[171,181]
NA	V118 (of unknown genetic background)	0.72 mg/g DW (whole fruit)	[183,184]
*Aft*/*Aft atv*/*atv*	Japanese blue tomato (a cv. of ‘Indigo Rose’)	17 mg/g DW (peel extract); 0.1 mg/g DW (flesh extract)	[133]
*Aft*/*Aft atv*/*atv;* NA	Black cherry tomato cultivars grown in Vietnam. (‘Indigo Rose’, ‘OG’, ‘F1:001’)	NA	[182]
*Aft*/*Aft atv*/*atv*	‘Sun Black’ (*Aft*, LA1996 × *atv*, LA0797)	1.2 mg/g DW (whole fruit); 0.07 mg/g FW (whole fruit)	[186]
*Aft*/*- hp-1*/*hp-1*	(LA1996, *Aft* × LA3538, *hp-1*)	0.9 mg/g FW (skin)	[19]
*Aft*/*Aft hp-1*/*hp-1*	(*Aft*, LA1996 × *hp-1hp-1,* Ailsa Craig)	NA	[172]
*Aft*/*Aft atv*/*atv hp2*/*hp2*	(‘Micro-Tom’)	NA	[187]
*Aft*/*Aft dg*/*dg*	(*AftAft*, Alisa Craig × *dgdg*, BCT-115)	0.21 mg/g FW (pigment-rich skin and pericarp)	[188]
*Aft*/*Aft atv*/*atv hp2*/*hp2*	(Commercial cherry tomato)	0.91 mg/g FW (peel)	[189]

Overall, the breeding efforts conducted so far have mainly exploited the synergistic effects of the two wild species alleles *Aft* and *atv* on anthocyanin accumulation in tomato fruit peel. Since 2011, when Myers released the first anthocyanin-rich, deeply purple-skinned variety, ‘Indigo Rose’, different commercial lines have been produced, likely carrying the same genetic combination.

The anthocyanins identified in the fruit peels of these (*Aft*/*Aft atv*/*atv*) breeding materials mainly belong to the same classes found in tomato vegetative tissues, i.e., petunidin, malvidin, and delphinidin, with petunidin-3-(*trans*-*p*-coumaroyl)-rutinoside-5-glucoside (petanin) being one of the most common and abundant (Table 3 and Table S2). Among the positive aspects of these breeding materials, anthocyanin accumulation in the fruit peel does not occur at the expense of other important tomato bioactive compounds, such as carotenoids [19,112,183,186]. Furthermore, the accumulation of anthocyanins in the skin of *Aft*/*Aft atv*/*atv* tomatoes was demonstrated to significantly extend their shelf life by delaying overripening and reducing susceptibility to the necrotrophic pathogen *Botrytis cinerea* [190].

The most intense and homogeneous accumulation of anthocyanins in the fruit peel of the purple *Aft*/*Aft atv*/*atv* tomatoes can be achieved only under strong light or low temperature conditions [170,179,180,185]. In contrast, high temperatures exert a negative effect on the anthocyanin content of these genotypes, and often, the pigmentation in the shaded area of the peel is not uniform [191]. Furthermore, because anthocyanin accumulation is limited to the fruit peel, the total amount of anthocyanins achieved in the whole fruit is not yet optimal and is inversely correlated with the size of the fruit [19,112]. Therefore, as recently highlighted by Menconi et al. [191], future breeding efforts aimed at increasing the anthocyanin content in tomato fruit should mainly try to activate the pathway also in the fruit flesh (a target that was achieved through genetic engineering, see Section 3.5), as well as improve the uniformity of pigmentation, making it a non-conditional phenotype.

### 3.5. Transgenic Approaches

Over the years, an increasing number of transgenic strategies have been implemented to induce the synthesis of anthocyanins and other flavonoid compounds in tomato fruit [154,155,192]. The main approaches employed initially were the overexpression of structural genes encoding biosynthetic enzymes (level-1 engineering) [119,193] or the use of transcription factors (TFs) to induce the activity of the secondary metabolic pathway (level-2 engineering) [77,118,131,194,195,196]. Due to poor knowledge of the regulators of the anthocyanin pathway in tomato, the majority of these early attempts have made use of heterologous genes. Although level-1 and level-2 engineering strategies can lead to high levels of secondary metabolites, the supply of precursors, energy, and reducing power from primary metabolism may still limit flux [154]. This constraint has been addressed with the discovery of a new type of transcriptional activator in plants, such as AtMYB12 [195], WRI1 [197,198], and GAME9 [199], that affect primary and intermediary metabolism (level-3 engineering) as well as specialized metabolism (level-2 engineering), thus adding a new strategy of engineering to the tomato toolbox [154,155,163]. In particular, the *SlE8:AtMYB12* line, expressing in a fruit-specific manner *AtMYB12*, a TF gene regulating flavonol biosynthesis in *Arabidopsis*, is considered a very important tool for metabolic engineering of the flavonoid pathway in tomato [155,163,195].

Considering that the *CHI* gene is expressed at a lower level than the other *EBG*s in the peel of tomato fruit, a first attempt to overcome this rate-limiting step was a constitutive expression of the petunia *CHI* gene [119]. The transgenic cv. FM6203 tomato lines had dramatic increases in fruit peel flavonols (up to a 78-fold increase in individual fruits) compared with control plants, mainly due to an accumulation of rutin (a quercetin glycoside) and smaller but still substantial increases in kaempferol glycosides. However, no anthocyanins were detected [119,200]. In addition, based on both metabolite and gene expression studies, Muir et al. [119] showed that the flavonoid biosynthesis pathway was not active in tomato fruit flesh. These findings indicated that a different strategy would be required to achieve upregulation of the entire flavonoid pathway in tomato fruit flesh [200].

With this goal in mind, Bovy et al. [131] developed transgenic FM6203 plants overexpressing, under the control of the fruit-specific E8 promotor, two anthocyanin biosynthetic regulatory genes of maize (*Zea mays* L.), the *Lc* (*Leaf color*; MYC-type) and *C1* (*colorless-1*; MYB-type), which were known to ectopically activate anthocyanin synthesis in other species. The results indicated that the expression of both TFs (*LC* and *C1*) was required and sufficient to activate the flavonoid pathway in tomato fruit flesh. However, anthocyanins could not be detected.

Partial successes in inducing anthocyanin production in cultivated tomato fruit were achieved through the overexpression of other MYB-type TFs, both endogenous (SlANT1; [77]) and heterologous (AtPAP1; [196]). However, in both cases, the transgenic tomato plants produced fruits with anthocyanin spots only in the peel tissue.

The first successfully engineered ‘purple’ tomatoes, with intense anthocyanin pigmentation of both peel and flesh, were produced by Butelli et al. [118]. These authors overexpressed two transcription factor-encoding genes of the anthocyanin pathway from snapdragon (*Antirrhinum majus* L.), *Delila* (*AmDel*; a bHLH TF), and *Rosea1* (*AmRos1*; an R2R3 MYB TF) in the cv. ‘Micro-Tom’ under the control of the tomato fruit-specific SlE8 promoter. Anthocyanin accumulation in these deeply purple tomatoes was uniformly distributed and light-independent, reaching contents of up to ~3 mg/g FW, which were comparable to those of blackberries and blueberries, and resulted in a significant increase in the fruit’s hydrophilic antioxidant capacity [118] (Table 4 and Table S3).

Notably, a preliminary study conducted on cancer-susceptible *Trp53*^−/−^ mice indicated that the *AmDel*/*AmRos1* tomatoes had enough anthocyanins to provide significant protection against the advancement of cancer when included in the mice’s regular diet **[118]**.

A detailed analysis of the phenolic content of the *AmDel*/*AmRos1* purple tomatoes, in comparison with the wild-type control, showed numerous metabolic alterations, including a reduction in rutin and naringenin chalcone, along with higher levels of anthocyanins and phenylacylated flavonol derivatives [201]. Interestingly, the high anthocyanin contents of the purple *AmDel*/*AmRos1* tomato fruits significantly extended their shelf life by retarding overripening and increasing resistance to gray mold (*Botrytis cinerea*) [202]. Several factors contributed to the success of the *AmDel*/*AmRos1* regulatory combination in tomato fruit (Section 3.6.1) [118,170].

Overall, these results highlight the importance of carefully selecting heterologous transcription factor(s) when pursuing genetic engineering aimed at modulating metabolite accumulation to ensure that the TFs have the appropriate specificity for their target genes in the selected crop species [118]. In addition, the use of the ethylene-inducible, fruit-specific promoter SlE8, which intervenes in the late stages of fruit ripening, generally allows the production of high levels of the desired metabolite without yield penalties or effects on plant growth. This might explain why the high anthocyanin production in these *AmDel*/*AmRos1* purple tomatoes was not associated with a reduction in other major classes of tomato fruit pigments, including carotenoids [118,170].

Following the successful results obtained in ‘Micro-Tom’, anthocyanin-rich *AmDel*/*AmRos1* purple tomatoes were obtained in other tomato varieties of commercial interest, such as ‘Arka Vikas’ [203], ‘Rubion’ [204,205], and ‘Moneymaker’ [206] (Table 4 and Table S3).

Tomato plants overexpressing the R2R3 MYB factor *ANT1* gene, isolated from both *S. chilense* (*ANT1^C^*) and *S. lycopersicum* (*ANT1^L^*), showed different purple pigment contents in the peel and flesh of the fruit. In particular, the accumulation of anthocyanins in the fruit flesh was greater in transgenic tomato lines expressing *ANT1^C^* [207]. The 35S promoter-driven expression of either *SlANT1* or its paralog *SlAN2* (another SG6 MYB gene) has been shown to be sufficient to induce anthocyanin production in all the main plant organs, including the flesh and the peel of the fruits [208]. Nevertheless, only *SlAN2* acted as a positive regulator of anthocyanin accumulation in vegetative tissues under high light or cold conditions [208]. These transgenic plants also showed greater tolerance to heat, cold, and oxidative stress as they kept ROS content low [209,210]. However, fruits overexpressing *SlAN2* were orange-colored, with a higher ethylene content and fast softening [211].

Recently, Jian et al. [132] have shown that overexpression of a single R2R3 MYB TF SlMYB75/SlAN2 can lead to abundant anthocyanin accumulation in both vegetative and reproductive organs, including seeds, stamens, and fruits, and this TF can be induced by various hormones or stress conditions. The anthocyanin content in the purple *SlMYB75-*OE ‘Micro-Tom’ fruits reached 1.86 mg/g FW, along with increased phenylpropanoid derivatives and aroma volatiles compared to the WT. *SlMYB75*-OE tomatoes also displayed a series of physiological changes, including increased ethylene production and delayed ripening. While in the previously reported transgenic *AmDel*/*AmRos1* purple tomatoes only delphinidin, petunidin, and malvidin classes were detected [118,201,205], in the purple *SlMYB75*-OE tomatoes six classes of anthocyanins (cyanidin, delphinidin, peonidin, petunidin, malvidin, and pelargonidin) were identified [212] (Table 4 and Table S3). Consistent with previous findings, the anthocyanin contents in the *SlMYB75*-OE fruits were positively correlated with higher antioxidant capacity, longer shelf life, and higher resistance to *B. cinerea* [212].

To investigate further the potential of using AtMYB12 to engineer phenylpropanoid metabolism in tomato based on a multi-level engineering approach, Zhang et al. [163] crossed the transgenic orange *SlE8:AtMYB12* tomato line [195] with the purple anthocyanin-enriched *AmDel*/*AmRos1* tomato line. The new hybrid tomato line was named ‘Indigo’ for its vivid blue–purple color, caused by the co-pigmentation of anthocyanins and high amounts of flavonols throughout the fruit. Compared with parental lines, ‘Indigo’ tomato fruit had even higher contents of the main phenylpropanoids anthocyanins (almost twice as high as the purple *AmDel*/*AmRos1* tomato), flavonols (3-fold more than *AtMYB12* tomatoes), and chlorogenic acid (CGA) (2-fold more than *AtMYB12* tomatoes) [163]. Interestingly, as mentioned above, this study indicated that *AtMYB12* regulates metabolism at multiple levels, as it can induce both primary metabolism (glycolysis, the TCA cycle, the pentose phosphate, and the shikimate pathways) and secondary metabolism.

Along these lines, Scarano et al. [213] obtained a ‘Bronze’ tomato by crossing the ‘Indigo’ tomato with a tomato line containing the *Stilbene Synthase* (*StSy)* gene from *Vitis vinifera,* which is involved in the production of resveratrol and stilbenoids. The ‘Bronze’ tomato, whose name refers to the metallic brown color of the skin, contained three different classes of polyphenols (flavonols, anthocyanins, and stilbenoids). However, the concentration of anthocyanins in ‘Bronze’ tomatoes was lower than that of the ‘Indigo’ parent (Table 4) [213]. On the other hand, the co-presence of the three different classes of polyphenols exerted synergistic effects for reducing bowel inflammation using a mouse model [213].

To further increase anthocyanin and flavonol levels in both the peel and the flesh of cv. ‘Rubion’ fruits, Lim and Li [214] introduced the *CHI* gene from onion, *Allium cepa* L*.,* into the *AmDel*/*AmRos1*-expressing tomatoes. The *CHI*/*AmDel*/*AmRos1*-expressing lines had significantly higher anthocyanin content than the *CHI*- or *AmDel*/*AmRos1*-only transgenic tomatoes in both the peel and the flesh (Table 4). For instance, for *CHI*/*AmDel*/*AmRos1* transgenics, the authors reported up to 400-fold increases in the levels of anthocyanins in tomato peel compared with 100-fold increases in tomato peel observed in the *AmDel*/*AmRos1*-only-expressing lines.

Another interesting result was obtained by Sun et al. [152]. The authors engineered, in the cultivar ‘Ailsa Craig’, the expression of the master regulator *SlAN2-like^InR^* under the control of the fruit-specific SlE8 promoter with the aim of bypassing the requirement for light and activating anthocyanin biosynthesis in the fruit flesh of any cultivar. As hypothesized by the authors, the resulting *proSlE8:SlAN2-like^InR^* plants exhibited, in a light-independent manner, anthocyanin accumulation in both the peel and flesh [152].

In a recent study, Butelli et al. [215] successfully generated nearly isogenic tomato lines (NILs) with high levels of three subclasses of anthocyanins in the fruit flesh. To engineer the accumulation of novel anthocyanins in tomato fruit, the authors took advantage of the tomato natural mutant *anthocyaninless* (*a*), corresponding to *f3′5′h*, in which a premature stop codon prevents the production of F3′5′H and, therefore, the synthesis of anthocyanins [216]. This study showed that by combining fruit-specific engineering (under the control of the SlE8 promoter) of three regulatory genes (*AmDel*, *AmRos1*, and *AtMYB12*) and of a single biosynthetic gene (*AmDFR*) with the mutant (*f3′5′h)*, it was possible to expand the chemical diversity of anthocyanins to generate unique tomato NILs. The fruit flesh of each NIL produced high levels of one of the three subclasses of anthocyanins naturally found in strawberries, raspberries, and blueberries, namely, pelargonidin (orange/red hue), cyanidin (red/magenta hue), or delphinidin (violet/blue or magenta/purple)-based derivatives [4,215]. This was highlighted by the authors as the first instance of nearly isogenic plant material, each containing specific subclasses of anthocyanins.

**Table 4:** Genetic engineering achievements to induce/improve tomato fruit anthocyanin content. Tomato lines, *S. lycopersicum* genetic background, total anthocyanin content. Abbreviations: FW, fresh weight; DW, dry weight; WT, wild type; NA, not available.

Tomato Line	*S. lycopersicum* Genetic Background	Total Anthocyanin Content	Reference
‘Purple’ (*SlE8::AmDel*/*AmRos1*)	cv. ‘Micro-Tom’	2.83 mg/g FW (whole fruit)	[118]
‘Purple’ (*SlE8::AmDel*/*AmRos1*)	cv. ‘Arka Vikas’	0.13 mg/g FW (pulp and skin)	[203]
‘Purple’ (*SlE8::AmDelAm*/*Ros1*)	cv. ‘Rubion’	5.1 mg/g DW (peel) 5.8 mg/g DW (flesh) 5.2 mg/g DW (whole fruit)	[204,205]
‘Purple’ *(SlE8::AmDel*/*AmRos1)*: ‘Micro-Tom’ *AmDel*/*AmRos1* × ‘Moneymaker’	cv. ‘Moneymaker’	3 mg/g DW (whole fruit)	[206]
‘Purple’ (*SlMYB75*/*SlAN2-OE*)	cv. ‘Micro-Tom’	1.86 mg/g FW (whole fruit)	[132]
‘Indigo’ (*SlE8:AmDel*/*AmRos1* × *SlE8:AtMYB12*)	cv. ‘Micro-Tom’	ca. 5.5 mg/g DW (whole fruit pericarp)	[163]
‘Bronze’ *(SlE8*:*MYB12*, *SlE8*:*AmDel*/*Am*/*Ros1*, 35S:*StSy*): ‘ResTom’ (*SlE8:MYB12*, *35S:StSy*) × ‘Indigo’ (*SlE8:MYB12*, *E8:AmDel*/*AmRos1*)	NA	‘Bronze’: ca. 2.5 mg/g DW (whole fruit) ‘Indigo’: ca. 5.0 mg/g DW (whole fruit)	[213]
*CHI*/*AmDel*/*AmRos1*: (*CHI* × *AmDel*/*AmRos1 lines*)	cv. ‘Rubion’	*CHI*/*Del*/*Ros1*: 3.25 mg/g FW (peel) *Del*/*Ros1*: 0.8 mg/g FW (peel) WT: 0.008 mg/g FW (peel) *CHI*/*Del*/*Ros1*: ca. 0.31 mg/g FW (flesh) *Del*/*Ros1*: ca. 0.01 mg/g FW (flesh) WT: ca. 0.001 mg/g FW (flesh)	[214]
*proSlE8:SlAN2-like^InR^*	cv. ‘Ailsa Criag’	up to 2.22 mg/g FW (flesh)	[152]
‘Purple’ (*AmDel*/*AmRos1*)	cv. ‘Micro-Tom’	14.7 mg/g DW (whole fruit)	[215]
‘Pink’ (*AmDel*/*AmRos1; f3′f’h*)	‘Micro-Tom-like’	1 mg/g DW (whole fruit)	[215]
‘Crimson’ (*AmDel*/*AmRos1; AmDFR; f3′5′h*)	‘Micro-Tom-like’	5.3 mg/g DW (whole fruit)	[215]
‘Magenta’ (*AmDel*/*AmRos1; AmDFR; AtMYB12; f3′5′h*)	cv. ‘Micro-Tom-like’	7.9 mg/g DW (whole fruit)	[215]
‘Indigo’ (*AmDel*/*AmRos1; AtMYB12*)	cv. ‘Micro-Tom’	24.7 mg/g DW (whole fruit)	[215]

In addition to traditional breeding and metabolic engineering approaches, “New Genomic Techniques” (NGTs) or “Genome Editing” have provided very efficient tools for crop improvement, although they are also challenged by varying and evolving restrictions and consumers’ concerns worldwide [217]. CRISPR/Cas9-mediated gene editing has been successfully applied in tomato. Numerous genes underlying traits of interest have been edited and functionally characterized using this technology [218]. As regards the anthocyanin pathway, the CRISPR/Cas9 system has been mainly used to confirm the function of several TFs (e.g., AN2, ANT1, ANT1-like, AN2-like, SlMYBATV, and HY5) (see Section 3.6.2) [151,152,180,219]. In particular, CRISPR/Cas9-induced mutations of the R3 MYB repressor gene *SlMYBATV* in the cultivar LA1996 (*AftAft*) were able to restore the stronger anthocyanin pigmentation phenotype of ‘IndigoRose’ (*AftAft atvatv*) fruit peel.

**Figure 4:**
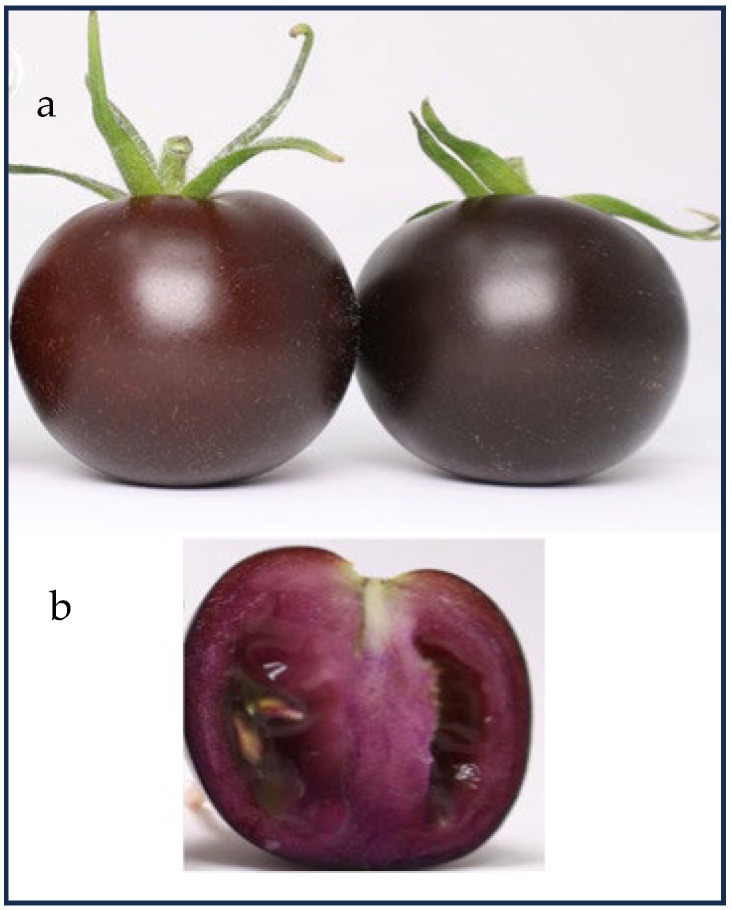
Transgenic purple tomato fruits. Whole fruit at Breaker (B) + 7 days (left) and B + 9 days (right) (**a**) and transverse section of fruit at B + 9 days (**b**). Photographs provided by Andrew Horgan, Texas A&M University.

Overall, transgenic approaches have demonstrated that, if properly activated, the anthocyanin biosynthetic pathway is fully present and functional in tomato fruit. Compared to traditional breeding (Section 3.4), the “purple” tomato lines produced by genetic engineering are characterized by light-independent, uniform, deeply purple fruits with high anthocyanin concentrations in both peel and flesh (Figure 4) [118]. The main anthocyanins identified in the ‘Purple Tomato’ *Del*/*Ros1* lines were delphinidin-based petanin and nasunin (Table 4 and Table S3). The transgenic approaches have also expanded the chemical diversity of anthocyanins in tomato fruit to include pelargonidin- and cyanidin-based derivatives [215].

In spite of a general consumer reluctance to accept genetically modified (GM) food, the genetically engineered *Del*/*Ros1*-N ‘Purple Tomato’ [118] was recently approved by the US Department of Agriculture/Animal and Plant Health Inspection Service (USDA/APHIS) in September 2022 (https://www.aphis.usda.gov/aphis/newsroom/stakeholder-info/sa_by_date/sa-2022/purple-tomato; accessed on 19 March 2024). In July 2023, it received the FDA notification (www.fda.gov/media/170056/download, accessed on 6 June 2024), and now it can be found in markets in the USA (Figure 4).

### 3.6. Genetic Regulation of the Pathway

In tomato, the availability of mutants obtained through both classical breeding and genetic engineering has allowed us to understand the anthocyanin biosynthetic pathway and the complex transcriptional network that regulates its expression. This network involves various transcription factors that either activate biosynthesis, such as MYB, basic bHLH, and WDR TFs, or repress it, as the transcription factors R3-MYB and R2R3-MYB [191,192].

To identify candidate genes that may correspond to loci influencing anthocyanin accumulation in tomato, eggplant, potato (*Solanum tuberosum* L.), and pepper (*Capsicum* spp.), De Jong et al. [216] localized thirteen genes known to be involved in anthocyanin biosynthesis (*chs*, *chi*, *f3h*, *dfr*, *f3′5′h*, *ans*, *3gt*, *rt, fsl)* or its regulation (*an1*, *an2*, *an11*, *jaf13*) in petunia on a tomato molecular genetic map. Several classical tomato mutants affecting anthocyanin accumulation had previously been described and mapped on the tomato high-density RLFP linkage map [220]. Similar map positions suggested that the genes flavonoid 3′5′-hydroxylase (*f3′5′h*), anthocyanidin synthase (*ans*), and the petunia Myb domain transcriptional regulatory gene *an2* might correspond to the tomato mutants *anthocyaninless* (*a*), *entirely anthocyaninless* (*ae*), and *anthocyanin gainer* (*ag*), respectively [216]. Although this study allowed the identification of the major genes involved in the anthocyanin biosynthetic pathway, knowledge about their genetic regulation was still very limited.

Subsequently, classical breeding of the tomato *Aft*/*Aft atv*/*atv* (e.g., ‘Indigo Rose’) and *Abg*/*-atv*/*atv* lines, showing strong anthocyanin pigment accumulation in the fruit peel under high light conditions (see Section 3.4 [19,170,171]), stimulated much research aimed at cloning the genes underlying the *Aft*, *atv*, and *Abg* loci (see Section 3.6.2). This work also allowed the elucidation of the regulatory network of the anthocyanin pathway in tomato [191,192].

#### 3.6.1. Biosynthetic Pathway Genes

Transcript profiling analysis using qRT-PCR and microarray data allowed the identification of anthocyanin biosynthetic genes differentially expressed in the fruit peel of the *Aft*/*Aft atv*/*atv* genotype, which has intense purple fruit peel, in comparison to Ailsa Craig, *Aft*/*Aft*, and atv/atv [179]. Both EBGs and LBGs were included in the analyses. The results showed that anthocyanin levels and the expression of the genes involved in anthocyanin production and compartmentalization (*F3′5H, DFR, ANS, AAC, 3GT, PAT*, and *GST*) were strongly upregulated in the *Aft*/*Aft atv*/*atv* line, compared to the individual parental lines. Furthermore, the results showed that the two alleles, *Aft* and *Atv*, had a synergistic effect on the transcription of specific anthocyanin genes and that this resulted in the activation of the entire anthocyanin pathway.

Kang et al. [221] evaluated the expression profiles of key anthocyanin biosynthetic genes in seven tomato genotypes with distinct fruit colors (red, pink, orange, yellow, green, brown, and purple). In line with the results reported by [179], transcript qRT-PCR data revealed that both EBGs and LBGs of the anthocyanin pathway were upregulated in the peels of purple tomato fruits (except for the *Sl5GT* gene) compared to any other tomato lines analyzed. 

As for the genetically engineered purple tomatoes, transcript profiling showed that fruit-specific expression of the two TF genes *AmDe*l and *AmRos1* induced the transcription of almost all the enzyme-encoding genes involved in flavonoid/anthocyanin biosynthesis, some genes involved in the anthocyanin side-chain modification, and two genes possibly related to final transport into the vacuole [118,170]. Among the structural genes of the phenylpropanoid/flavonoid pathway activated by *AmDel*/*AmRos1*, worthy of note are *PAL*, *CHI,* and *F3′5′H*, which strongly promote flavonoid biosynthesis. Interestingly, all three genes were not induced when the maize *Lc*/*C1* regulatory combination was expressed in tomato plants (which led to higher levels of flavonols but no anthocyanins in the fruits) [131]. While *CHI* and *F3′5′H* are both critical to direct the flux of flavonoid intermediates towards the anthocyanin products, *PAL* induction is required to guarantee high levels of flux through general phenylpropanoid metabolism to feed flavonoid biosynthesis [170]. Furthermore, the different outcomes resulting from the ectopic expression of *AmDel*/*AmRos1* vs. *Lc*/*C1* in tomato confirmed the key role played by the *F3′5′H* structural gene in the activation of anthocyanin synthesis in tomatoes [118].

#### 3.6.2. MYB Transcription Factors

Among the three wild allelic variants (*Atv*, *Abg,* and *atv*) used in breeding to induce anthocyanin accumulation in cultivated tomato fruit, the *atv* locus was the first for which the molecular basis was identified [148,149]. Cao et al. [148] fine-mapped the *atv* locus to a small interval on the long arm of chromosome 7, harboring only one gene, *Solyc07g052490*, putatively encoding an R3 MYB repressor, which was thus named *SlMYB-ATV*. In the *atv* genotype, *SlMYB-ATV* is mutated and non-functional due to a 4-bp insertion in its coding region that leads to a truncated protein. In the *Aft*/*Aft atv*/*atv* genotypes, the mutated Slmyb-atv protein was related to higher expression of the anthocyanin structural genes and a more intense anthocyanin pigmentation in the fruit peel, compared to the *Aft*/*Aft ATV*/*ATV* line. These results were confirmed by Colanero et al. [149] through genome sequencing of the *atv* line, followed by candidate gene analysis. In wild-type plants, the functional SlMYB-ATV protein directly binds the bHLH tomato factors involved in MBW complexes, acting as a repressor of the MBW complex. Notably, transcriptional activation of *SlMYB-ATV* was by the same MBW complex as previously proposed in the model of the regulation of the anthocyanin pathway in eudicots [222] and, at the same time, under its own repression. This generates a feedback inhibition that fine-tunes biosynthesis to avoid excessive anthocyanin accumulation.

Through a genome-wide search, Cao et al. [148] identified another two candidate R3 MYB repressors (SlMYBATV-like and SlTRY) and four candidate R2R3 MYB repressors, SlMYB3, SlMYB7, SlMYB32, and SlMYB76. Recently, Zhang et al. [223] found that *SlMYB7* expression was positively correlated with the anthocyanin content changes observed in ‘Black Pearl’ fruit, which are purple–green, then become dark purple as they ripen, and finally turn purple–red when fully ripe. Transient overexpression revealed that SlMYB7 acted as a repressor of anthocyanin synthesis by inhibiting the transcription of structural genes of the pathway through direct promoter binding and interacting with the bHLH proteins SlJAF13 and SlAN1, thus precluding the formation of the MBW complex.

At the *Aft* locus, a cluster of four highly homologous anthocyanin-related R2R3 MYB TF-encoding genes, namely *Solyc10g086260* (*SlANT1*) [77,172,179,207,208], *Solyc10g086250* (*SlAN2*/*SlMYB75*) [208,216,224,225], *Solyc10g086270* (*SlANT1-like*/*SlMYB28*) [179,208,225], and *Solyc10g086290* (*SlAN2-like, SlMYB114*) [225], was identified on the distal end of the long arm of chromosome 10 [192]. The four MYB TF genes are located close to each other, suggesting that a tandem gene duplication event may have given rise to this cluster of genes [151,226]. *SlANT1* was the earliest R2R3-MYB TF gene described in tomato to be clearly involved in anthocyanin biosynthesis, and it was identified using an activation-tagging strategy [77]. *SlANT1* and its paralog *SlAN2* were the first two R2R3 MYB TF-encoding genes to be considered as candidates controlling anthocyanin and flavonol accumulation in fruit peels of the *Aft* genotype [172,196,207,224,225]. Furthermore, constitutive overexpression of either *SlANT1* or *SlAN2* demonstrated that they both are functional activators of the pathway [208]. However, only SlAN2 is known to be the principal R2R3 MYB TF implicated in the MBW complex in tomato vegetative tissues via regulating the expression of the bHLH TFs SlAN1 and SlJAF13 [192,208,219].

Three recent independent studies, using the purple tomato cultivar ‘Indigo Rose’ and/or the *Aft* accession LA1996, have finally revealed the genetic basis of the *Aft* locus as the R2R3 MYB encoding gene*, SlAN2-like*, that positively regulates anthocyanin production in tomato fruits [150,151,152]. These results definitively ruled out the other R2R3 MYB-encoding genes (*SlANT1, SlAN2,* and *SlANT-1like*) in the *Aft* introgressed region as candidates. These studies employed different but complementary approaches, which allowed not only the identification of the causal gene of *Aft* but also provided the complete mechanism for how *Aft*/*Aft atv*/*atv* synthesizes anthocyanins in the fruit peel in a light-dependent manner [192,227].

In particular, Sun et al. [152], aside from confirming many of the results reported by Colanero et al. [150] and Yan et al. [151], added further information. By using mainly the CRISPR/Cas9 gene editing system, they managed to reconstruct the whole gene network that drives anthocyanin accumulation in the fruits of the double mutant *AftAft atvatv* ‘Indigo Rose’ [192]. In line with a previous report by Qiu et al. [180], Sun et al. [152] showed that the bZIP transcription factor SlHY5 is a key anthocyanin regulator in light-exposed fruit peels of ‘Indigo Rose’ and further demonstrated that the promoter of *SlAN2-like^InR^* is a direct transcriptional target of SlHY5. The bHLH transcription factor SlAN1, known to positively regulate anthocyanin biosynthesis in tomato seedlings [147], was confirmed to promote anthocyanin accumulation also in the ‘Indigo Rose’ fruit peel. In addition, Sun et al. [152] proved that the functional *SlAN2-like^InR^* allele activates the expression of both the positive regulator SlAN1 (bHLH), with which it can form the key MBW complex of tomato that induces the expression of anthocyanin structural genes, and the negative regulator *SlMYB-ATV* (R3-MYB), whose protein, in turn, can compete with SlAN2-like^InR^ in binding the bHLH SlAN1, thus contributing to a negative feedback loop that fine-tunes anthocyanin accumulation [149]. The non-functional *SlAN2-like* allele is caused by a splicing mutation leading to the production of a truncated protein that is unable to interact with the bHLH and WDR partners of the MBW anthocyanin complex [150,152]. Based on all these findings, Sun et al. [152] suggested that *SlAN2-like^InR^* functions as a master regulator in the activation of anthocyanin biosynthesis and that this key TF could be engineered to trigger anthocyanin production in the fruit flesh of cultivated tomatoes in a light-independent manner. The authors confirmed this hypothesis by expressing the functional *SlAN2-like^InR^* allele under the control of the fruit-specific SlE8 promoter, which led to fruits with increased anthocyanin accumulation in both peel and flesh.

Another allele of *SlAN2-like* has recently been identified by Fenstemaker et al. [137] in the tomato wild relative *S. galapagense*. This species belongs to the red and yellow clade, which is very close to *S. lycopersicum*. Nevertheless, the *S. galapagense* accession LA1141 shows an anthocyanin-based purple fruit pigmentation previously described in green-fruited wild tomatoes. QTL analysis in a LA1141 × cv. OH8245 introgression population identified three QTLs associated with the LA1141 purple color of the fruit, corresponding to the loci *atv* on chromosome 7, *Aft* on chromosome 10 (long arm), and *u* also on chromosome 10 (short arm). Sequence analysis suggested that the regulatory mechanism underlying the purple pigmentation of LA1141 fruit might be the same as the one described in the green-fruited tomato accessions. However, the LA1141 alleles of *Aft* and *atv* differ from those previously described in the green-fruited donor species. Interestingly, phylogenetic analysis of the *Aft* sequence suggested that the purple fruit pigmentation in this *S. galapagense* accession may have originated from a gain-of-function mutation that restored *Aft* [137].

The recent publication of a chromosome-scale, reference genome, and RNA-Seq data for *S. lycopersicoides* LA2951 by Powell et al. [161] pointed to *SlydAN2-like* as the best candidate for the *Abg* purple fruit phenotype. Subsequently, Menconi et al. [153] showed that the *AN2-like^Abg^* allele has the same SNP in the second intron as *AN2-like^Aft^*. The role of the gene in the phenotype was validated through *SlAN2-like* silencing in *Abg* fruits [153]. However, unlike *AN2-like^Aft^*, the *AN2-like^Abg^* allele can be alternatively spliced, resulting in two transcripts of different lengths and activities. The shorter transcript is much less expressed than the longer transcript and encodes a protein that is less efficient at transcriptional activation. As the authors highlight, this is the first report of a mechanism of alternative splicing in an R2R3 MYB TF gene, which may represent a new regulatory system of the anthocyanin pathway in tomato fruit peel.

The very low anthocyanin levels observed in the vegetative tissues of *Abg*/*Abg* plants seem to be mainly caused by the very weak expression of *AN2* [153]. Finally, a novel gene belonging to the *R2R3 MYB* cluster was identified at the *Abg* locus, which was named *MYB113^Abg^* for its similarity with other Solanaceae genes [82,153,228]. This gene was also found by Powell et al. [161], and it was named *AN2-like2*. The *MYB113^Abg^* gene encodes another potential positive regulator of anthocyanin biosynthesis; however, the *MYB113^Abg^* ortholog was mutated and non-functional in tomato and in several close relatives [153].

Based on the results reported so far, the anthocyanin locus lying in the distal end of the long arm of tomato chromosome 10 harbors a cluster of six anthocyanin-related R2R3-MYB TFs genes, five of which are activators, namely *SlANT1, SlAN2, SlANT1-like, SlAN2-like, SlAN2like-2*/*SlMYB113*, and one *SlMYB32*/*SlTHM27* is a repressor [153,191,192,208]. In *Abg,* all five activator genes produce functional TFs; in *Aft,* the first four are functional, whereas in WT tomato, only the first three activator genes are functional [191]. There is a high degree of synteny for this locus across the four Solanaceous crops: tomato, eggplant [48,52,61], potato [229], and pepper [230], and, as described in Section 2.3, major QTLs underlying the majority of the phenotypic variation in fruit and leaf anthocyanin pigmentation in eggplant were mapped on chromosome 10 [48,49,52,54,57].

Two additional MYB-encoding genes, *SlMYB7-like* and *SlMYB48-like,* have been identified by Jia et al. [231] as possible positive regulators of anthocyanin synthesis in tomato. Interestingly, both genes are targets of miR858, which acts as a negative regulator of the same pathway [191].

#### 3.6.3. bHLH Transcription Factors

As previously described, in addition to MYB transcription factors, another fundamental component of the MBW ternary complex is represented by proteins with basic helix–loop–helix (bHLH) motifs. Kiferle et al. [208] identified two bHLH factors involved in the anthocyanin pathway, named SlAN1 and SlJAF13 (also known as SlGL3*,* Colanero et al. [192]), for their strong homology to PhAN1 and PhJAF13, respectively. The two TFs are encoded by the genes *Solyc09g065100* and *Solyc08g081140,* which likely correspond to the sequences mapped by De Jong et al. [216] on chromosomes 9 and 8 of tomato, respectively. Hitherto, SlAN1 and SlJAF13 were the only bHLHs known to be involved in the Solanaceae MBW complex [191]. Subsequently, through map-based cloning, *SlAN1* was identified by Qiu et al. [147] as the mutated gene underlying the well-known *Hoffman’s anthocyaninless* (*ah*) locus and named *AH* (also known as SlTT8, Colanero et al. [192]). Transcriptomic analyses demonstrated that AH acts as a key transcriptional regulator of anthocyanin biosynthesis in tomato. Recently, Chen et al. [232], using an ethyl methanesulfonate (EMS)-mutagenized population of *Aft* (LA1996), have demonstrated that *SlJAF13* is the causal gene for an anthocyanin-deficient tomato fruit mutant *at4*. Interestingly, the results suggested that SlJAF13 acts as a dual-function transcription factor that fine-tunes anthocyanin biosynthesis and accumulation in the *Aft* tomato through its involvement in two different pathways.

#### 3.6.4. WD40 Transcription Factors

The third fundamental element of the MBW complex is represented by the WD-repeat proteins. Kiferle et al. [208] identified a tomato anthocyanin-related WDR protein, encoded by the gene *Solyc03g097340*, by homology with petunia PhAN11, which was therefore named SlAN11. This gene likely corresponds to the sequence mapped by De Jong et al. [216] on chromosome 3 of tomato. Subsequently, Gao et al. [233] characterized the molecular function of SlAN11 and showed that it participates in flavonoid biosynthesis in both tomato plants and seeds. Through yeast two-hybrid assays, it was proven that SlAN11 interacts only with bHLH TFs, including SlTT8 (or SlAN1/AH) and SlGL3 (or SlJAF13), but not with MYB proteins in the ternary MBW complex. 

Taken together, the results obtained so far in tomato support the models proposed by Albert et al. [222] and Montefiori et al. [234], in which a hierarchical mechanism seems to control the role of bHLH factors **[191]**. In fact, in tomato and, more generally, in the Solanaceae, two different complexes have been identified. The first one, created by an R2R3-MYB (e.g., SlAN2like in tomato fruit or SlAN2 in tomato vegetative tissues), the bHLH1 factor SlJAF13, and the WDR protein SlAN11 [151,232], induces the expression of the second factor bHLH2 gene, *SlAN1,* which is essential for anthocyanin biosynthesis in tomatoes [147]. SlAN1, along with the R2R3-MYB proteins and the WDR protein SlAN11, take part in the second complex that induces the expression of SlAN1 (bHLH2) (reinforcement) and anthocyanin biosynthesis genes, leading to anthocyanin accumulation [192,234]. R3-MYB repressors, such as SlMYB-ATV, ensure feedback inhibition. They are triggered by the MBW complex and prevent new MBW complexes from forming by titrating bHLH factors.

#### 3.6.5. Other Transcription Factors

Besides the members of the MBW ternary complexes, other TF families, such as HY5, BBX, PIF, WRKY, AREB, HD-bZIP, and NAC, are also involved in the regulation of anthocyanin biosynthesis, especially under environmental stimuli (including light, low temperature, and phosphorus deficiency stresses) or phytohormones [152,180,235,236,237,238,239]. In particular, recently, Luo et al. [235] showed that the photomorphogenesis-related transcription factor SlBBX20 interacts with the COP9 signalosome subunit SlCSN5-2 and enhances anthocyanin biosynthesis in several tomato tissues by directly binding the promoter of the *SlDFR* gene.

In tomato fruits, anthocyanin production is completely dependent on light. The role of the bZIP photomorphogenesis-promoting transcription factor HY5 as a positive, light-dependent regulator of anthocyanin synthesis has been demonstrated in the purple-skinned tomato accassion ‘Indigo Rose’ [152,180]. However, consistent with the results reported in other plant species, *Slhy5* mutants still accumulate residual anthocyanins in the fruit peel under high light [180]. Transcriptome analysis of several tissues of the knockout *Slhy5* mutants in the ‘Indigo Rose’ background allowed the identification of candidate TFs belonging to different families, such as HD-bZIP, WRKY, NAC, and PIF, which probably play roles in regulating anthocyanin synthesis in tomatoes in an HY5-independent manner, under high-light [180,240]. Notably, through yeast two-hybrid (Y2H) assays, He et al. [240] showed that SlBBX24, SlWRKY44, and, unexpectedly, two PIF TFs, SlPIF1 and SlPIF3, physically interacted with some of the TFs contributing to the formation of the MBW complex. Overall, their findings led the authors to speculate on the possible involvement of *SlPIF1*, *SlPIF3*, *SlBBX24*, and *SlWRKY44* in anthocyanin biosynthesis in a SlHY5-independent or dependent manner.

As in eggplant, the regulatory mechanism of anthocyanin biosynthesis in tomato is complex and needs further investigation. However, transcription factors remain the main target to increase the anthocyanin content in the fruit through the application of traditional breeding approaches, transgenic strategies, or modern genome editing technologies.

## 4. Conclusions

Anthocyanins are a diverse group of plant secondary metabolite pigments with roles in reproduction, dispersal, and protection from abiotic and biotic stresses. In addition to providing color to our fruits and vegetables, anthocyanins are strong antioxidants with numerous reported health benefits. Analyses of a range of eggplant cultivars and tomato mutants have revealed that genotypes of both species can express widely variable amounts and types of anthocyanins, differences that are not apparent in the fruit’s visible phenotype. Thus, the examination of additional genotypes in both species will undoubtedly yield an expanding list of anthocyanin compounds that should be studied for their specific bioactivity. Identification of new health-promoting compounds then opens the door to breeding cultivars with unique functional food properties.

Although the biosynthetic pathway of anthocyanin synthesis is conserved in eggplant and tomato, breeding is complicated by the fact that allelic differences in the early and late biosynthesis structural genes can result in distinct anthocyanin profiles. An additional layer of complexity is added by the regulatory genes, such as those that code for the MBW–TF complex, as well as noncoding RNAs and other epigenetic factors. Many different TFs with effects on the up or downregulation of anthocyanin biosynthesis have been identified in eggplant and tomato; however, more research is needed to determine to what extent these TFs are conserved across species.

Genetic engineering and, more recently, gene editing have proven to be excellent methods for exploring the intricacies of anthocyanin biosynthesis in both eggplant and tomato. The continuation of such research will allow the development of a comprehensive model for the control of anthocyanin accumulation in solanaceous species. This knowledge can be leveraged by breeders to offer farmers and consumers a wider range of anthocyanin-rich products from these species. The use of directed strategies like gene editing for modifying anthocyanin content will also help to minimize potential negative effects while providing transgene-free plants. Several studies have indicated that increased anthocyanin content in both eggplant and tomato can have benefits for plant health, such as increased tolerance to drought, freezing, and salinity, longer shelf life, and fungal disease resistance. While these effects may be related to the antioxidant activity of anthocyanins, the exact mechanisms are unknown and must be more thoroughly studied. Such research will benefit farmers and consumers who are conscious of the effects of climate change on plant health and food production and, in turn, the effects of agriculture on the environment.

## Figures and Tables

**Table 1:** Anthocyanin QTLs and candidate genes on chromosome 5.

Anthocyanin Trait	Location	QTL Name	Position (cM)	Candidate Gene	Reference
stem	E05	*steanE05.ML,MT*	69.73		[49]
	E05	*stean*	94.93		[51]
	E05	*stean5.1 BT,ML*	66.39	*HSP*,*BHLH93*,*AAT*	[54]
	E05	*stean5.1 MT*	64.51	*HSP*,*BHLH93*,*AAT*	[54]
hypocotyl	E05	*hyan5.1*	75.48	*CML*,*BKI1*,*TOGT1*,*BHLH8*,*AZF3,ZAT1*0,*CYP81Q32*,*VQ31*	[54]
leaf vein	E05	*lveanE05.ML,MT*	75.30		[49]
	E05	*adlvean*	87.34		[51]
peduncle	E05	*pedanE05.ML*	59.81		[49]
	E05	*pedanE05.MT*	75.30	*5GT*	[49]
	E05	*pedan*	87.34		[51]
calyx	E05	*calanE05.ML*	75.30	*5GT*	[49]
	E05	*calanE05.MT*	59.81		[49]
corolla	E05	*corcolE05.ML,MT*	75.30	*5GT*	[49]
	E05	*ca5.1*	73.3/T0612		[52]
	E05	*corcol5.1 BT,ML*	75.48	*CML*,*BKI1*,*TOGT1*,*BHLH8*,*AZF3,ZAT1*0,*CYP81Q32*,*VQ31*	[54]
	E05	*corcol5.1MT*	39.39		[54]
fruit	E05	*fcol*	100.27		[51]
	E05	*FrucolE05.ML*	75.304		[53]
	E05	*fap*	44.2		[50]
under calyx	E05	*UndcalE05.ML,MT*	75.304		[53]
D3R	E05	*D3RE05.ML,MT*	75.304		[53]
	E05	*P-D3R5.1*	66.4		[56]
nasunin	E05	*NasE05.ML,MT*	75.304		[53]
	E05	*P-NAN5.1*	66.4	*CRY1*,*BEAT*	[56]
	E05	*d3g*	80	*3GT*	[59]

**Table 2:** Anthocyanin QTLs and candidate genes on chromosome 10.

Anthocyanin Trait	Location	QTL Name	Position (cM)	Position (Mb)	Candidate Gene	Reference
stem	E10.1	*steanE10.ML,MT*	68.92		*CHS*	[49]
	E10.2	*sa10.1*	109.8/CT240			[52]
	E10.2	*sa10.2*	121.2/CT124			[52]
	E10.1	*stean*	69.13, 69.39, 70.39			[51]
	E10.2	*stean*	128.3, 128.34, 128.55			[51]
	E10.3	*stean10.1 BT,ML,MT*	231.46	94.7	*BES1*/*BZR1*,*DREB2C*,*PPC6-1*,*RAPTOR*	[54]
hypocotyl	E10.3	*hyan10.1*	231.46	94.7	*BES1*/*BZR1*,*DREB2C*,*PPC6-1*,*RAPTOR*	[54]
prickle	E10.2	*pa10.1*	109.2/.8/CT214-CT240			[52]
	E10.1	*lpc10.1*	79.02–79.94	87.85–95.97		[55]
leaf	E10.1	*adlanE10.ML,MT*	69.39		*3GT*,*AN1*,*ANT2*	[49]
	E10.1	*ablanE10a.ML*	68.92		*3GT*,*AN1*,*ANT2*	[49]
	E10.1	*ablanE10.MT*	68.58		*3GT*,*AN1*,*ANT2*	[49]
	E10.2	*lla10.1*	109.8/TG233-TG63			[52]
	E10.1	*adlan*	69.13			[51]
	E10.1	*adlan*	69.39			[51]
	E10.3	*adlan10.1BT,ML,MT*	236.98	95	peroxidase, *PDI*	[54]
leaf vein	E10.1	*lveanE10.ML*	69.13		*CHS*	[49]
	E10.1	*lveanE10.MT*	68.92		*CHS*	[49]
	E10.1	*qLVC10*	63			[57]
	E10.2	*lra10.1*	110.5/TG233-TG63			[52]
	E10.1	*adlvean*	69.13			[51]
	E10.1	*adlvean*	69.39 (2)			[51]
	E10.2	*adlvean*	128.30, 128.34, 128.55			[51]
	E10.3	*lvean10.1 BT,ML,MT*	231.46	94.7	*BES1*/*BZR1*,*DREB2C*,*PPC6-1*,*RAPTOR*	[54]
	E10.1	*vc10.1*	79.44–80.14	85.12–95.97		[55]
peduncle	E10.1	*pedanE10.ML,MT*	69.13		*CHS*	[49]
	E10.1	*pedan*	69.13			[51]
	E10.1	*pedan*	69.39			[51]
	E10.2	*pedan*	128.30, 128.34, 128.55			[51]
calyx	E10.1	*calanE10.ML,MT*	68.92		*CHS*	[49]
		*calan*	6.25			[51]
	E10.1	*calan*	69.13, 69.39			[51]
	E10.1	*calan*	69.39			[51]
flower intensity	E10.3	*flian10.1 BT,ML,MT*	231.46	94.7	*BES1*/*BZR1*,*DREB2C*,*PPC6-1*,*RAPTOR*	[54]
corolla	E10.3	*corcol10.1 BT,ML*	232.77	94	*ANS*,*JRG21*,MYB-family TF,*SAC8*	[54]
fruit	E10.2	*fap10.1*	106.4/C2At3g08760			[52]
	E10.2	*fap10.2*	109.8/TG233-CT240			[52]
	E10.1	*fcol*	64.21			[51]
	E10.1	*fcol*	69.13			[51]
	E10.1	*fcol*	69.39			[51]
	E10.1	*qFPC10b*	63		predicted *WDR* gene	[57]
	E10.2	*fcol*	128.30, 128.34, 128.55			[51]
		*FA*		91.08–94.81	*MYB113*-like	[58]
under calyx	E10.1	*UndcalE10.ML,MT*	69.39			[53]
		*PUC10.2*		3.94–4.34	*COP1*	
		*PUC10.3*		91.08–94.81	*MYB113*-like	[58]
		*PUC10.4*		98.25–100.55		[58]
peel next to calyx	E10.1	*PnccE10.ML,MT*	69.39			[53]

## Supplementary Materials

The following supporting information can be downloaded at: https://www.mdpi.com/article/10.3390/ijms25126811/s1.
