# Supplementary material for: Genetic and Biotechnological Approaches to Improve Fruit Bioactive Content: A Focus on Eggplant and Tomato Anthocyanins
Source: Int J Mol Sci. 2024 Jun 20;25(12):6811. doi: 10.3390/ijms25126811 (PMC11204163; doi:10.3390/ijms25126811)
Supplement: Supplementary file 1 [file ijms-25-06811-s001.zip › Supplementary Table S2_Proofread_SUB.pdf]

Table S2. Breeding achievements to induce/improve tomato fruit anthocyanin content. Allelic combination of the main anthocyanin-enriched tomato lines, total anthocyanin content and the most abundant anthocyanins/anthocyanidins identified.

Abbreviations: Del, delphinidin; Pet, petunidin; Mal, malvidin; p-coum, para-coumaroyl; caf, caffeoyl; Cya, cyanidin; fer, feruloyl; rut, rutinoside; glc, glucoside; glyc, glycoside; hex, hexoside; arab, arabinose; FW, fresh weight; DW, dry weight. NA, not available.

Allelic Combination	Tomato Line (*S. lycopersicum* Genetic Background/ Original Cross)	Total Anthocyanin Content	Main Anthocyanins/ (Anthocyanidins) Identified	References
Aft/Aft	LA1996<br>(undeclared background)	0.66 mg/g FW (skin);<br>0.2 mg/g FW (pigment-rich pericarp beneath the skin)	(Petunidin**,** Malvidin and delphinidin)	[125]
Aft/Aft	LA1996<br>(undeclared background)	0.72 mg/g FW (skin) (2004);<br>0.18–0.36 mg/g FW (skin) (2006)	NA	[19]
Aft/Aft atv/atv	(*Aft*, LA1996 × *atv*, LA0797)	1.2 mg/g FW (skin)	Pet-3-(p-coum)-rut-5-glc	[19]
Abg- atv/atv	(*Abg* LA3668 × *atv*, LA0797)	4.2 mg/g FW (skin)	NA	[19]
Aft/Aft atv/atv	' Indigo Rose'	4.0 mg/g FW (peel extract);<br>0.09 mg/g FW (flesh extract)	Pet-3-(*trans*-p-coum)-rut-5-glc (petanin)<br>Mal-3-(*trans*-p-coum)-rut-5-glc (negretein)	[171, 181]
NA	V118<br>(of unknown genetic background)	0.72 mg/g DW (whole fruit)	Pet-3-(p-coum)-rut-5-glc;<br>Mal-3-(p-coum)-rut-5-glc;<br>Pet-3-caf-rut-5-glc	[183, 184]
Aft/Aft atv/atv	Japanese blue tomato<br>(a cv. of 'Indigo Rose')	17 mg/g DW (peel extract);<br>0.1mg/g DW (flesh extract)	Petunidin-derived anthocyanins	[133]
Aft/Aft atv/atv; NA	Black cherry tomato cultivars grown in Vietnam.<br>('Indigo Rose', 'OG', 'F1:001')	NA	Mal-3-(p-coum)-rut-5-glc (in IndR', 'OG', 'F1:001');<br>Pet-3-(fer)-rut-5-glc (in IndR', 'OG');<br>Del- 3-(p-coum)-rut-5-glc (in IndR', 'OG');	[182]

			Pet-3-(p-coum)-rut-5-glc (in IndR', 'OG', 'F1:001'); Del-3-(p-coum)-glc (in IndR', 'OG', 'F1:001'); Del-3-(p-coum)-glc+ arab (in IndR', 'OG', 'F1:001')	
Aft/Aft atv/atv	'Sun Black' (*Aft*, LA1996 × *atv*, LA0797)	1.2 mg/g DW (whole fruit); 0.07 mg/g FW (whole fruit)	Pet-3-(*trans*-p-coum)-rut-5-glc (petanin) Mal-3-(trans-p-coum)-rut-5-glc (negretein)	[186]
Aft/- hp-1/ hp-1	(LA1996, *Aft* × LA3538, *hp-1*)	0.9 mg/g FW (skin)		[19]
Aft/Aft hp-1/ hp-1	(*Aft*, LA1996 × *hp-1hp-1*, Ailsa Craig)	NA	(Petunidin, Malvidin and Delphinidin)	[172]
Aft/Aft atv/atv hp2/hp2	(cv. 'Micro-Tom')	NA	Delphinidin and Petunidin, mainly in acylated form	[187]
Aft/Aft dg/dg	(*AftAft*, Alisa Craig × *dgdg*, BCT-115)	0.21 mg/g FW (pigment-rich skin & pericarp)	(Petunidin, Malvidin and Delphinidin)	[188]
Aft/Aft atv/atv hp2/hp2	(Commercial cherry tomato)	0.91 mg/g FW (peel)	Pet-(p-coum)-rut-hex; Del-3-(p-couml)-rut-glyc ; Pet-(p-coum)-rut-hex ; Pet-3-(caf)-rut-5-glyc ; Mal-3-(p-coum)-rut-5-glyc Cya-3-O-rut	[189]
