# Supplementary material for: Genetic and Biotechnological Approaches to Improve Fruit Bioactive Content: A Focus on Eggplant and Tomato Anthocyanins
Source: Int J Mol Sci. 2024 Jun 20;25(12):6811. doi: 10.3390/ijms25126811 (PMC11204163; doi:10.3390/ijms25126811)
Supplement: Supplementary file 1 [file ijms-25-06811-s001.zip › Supplementary Table S3_Proofread_SUB.pdf]

Table S3. Genetic engineering achievements to induce/improve tomato fruit anthocyanin content. Tomato lines, *S. lycopersicum* genetic background, total anthocyanin content and the most abundant anthocyanins/anthocyanidins identified.

Abbreviations: Del, delphinidin; Pet, petunidin; Mal, malvidin; Cya, cyanidin; Pel, pelargonidin; Peo, peonidin; p-coum, para-coumaroyl; caf, caffeoyl; fer, feruloyl; rut, rutinoside; glc, glucoside; FW, fresh weight; DW, dry weight. WT, wild type; NA, not available.

Tomato Line	*S. lycopersicum* Genetic Background	Total Anthocyanin Content	Main Anthocyanins/ (Anthocyanidins) Identified	Referenc e
'Purple' (*SlE8::AmDel/AmRos1*)	cv. 'Micro-Tom'	2.83 mg / g FW (whole fruit)	Pet-3-(*trans*-p-coum)-rut-5-glc (petanin)<br>Del-3-(*trans*-p-coum)-rut-5-glc (nasunin)<br>Pet-3-(fer)-rut-5-glc;<br>Del-3-(fer)-rut-5-glc	[118]
'Purple' (*SlE8::AmDel/AmRos1*)	cv. 'Micro-Tom'	NA	Del-3–(*trans*-p–coum)-rut-5-glc (nasunin)<br>Pet-3–(*trans*-p-coum)-rut-5-glc (petanin)	[162]
'Purple' (*SlE8::AmDelAm/Ros1*)	cv. 'Arka Vikas'	0.13 mg/g FW (pulp and skin)	NA	[203]
'Purple' (*SlE8::AmDel/AmRos1*)	cv. 'Rubion'	5.1 mg /g DW (peel)<br>5.8 mg /g DW (flesh)<br>5.2 mg/g DW (whole fruit)	Del-3-(*trans*-p-coum)-rut-5-glc (Nasunin)<br>Pet-3-(*trans*-p-coum)-rut-5-glc (Petanin)	[204, 205]
'Purple' (*SlE8::AmDel/AmRos1*)<br>'Micro-Tom' Am*Del/AmRos1* × 'Moneymaker'	cv. 'Moneymaker'	3 mg /g DW (whole fruit)	NA	[206]
'Purple' (*SlMYB75/SlAN2-OE*)	cv. 'Micro-Tom'	1.86 mg/ g FW (whole fruit)	(Cyanidin, Delphinidin, Peonidin, Petunidin, Malvidin, Pelargonidin)	[132]
'Indigo' (*SlE8:AmDel/AmRos1* × *SlE8:AtMYB12*)	cv. 'Micro-Tom'	ca. 5.5 mg/ g DW (whole fruit pericarp)	Pet-(coum)- rut-glc<br>Del-(coum)- rut-glc<br>Pet-(fer)- rut-glc	[163]

			Mal-(coum)- rut-glc	
‘Bronze’(SlE8:*MYB12*, SlE8:*AmDel*/*AmRos1*, 35S:*StSy*):<br>‘ResTom’ (*SlE8:MYB12, 35S:StSy*) ×<br>‘Indigo’ (*SlE8:MYB12*, *E8:AmDel/AmRos1*)	cv. ‘Micro-Tom’	‘Bronze’: ca. 2.5 mg/ g DW (whole fruit)<br>‘Indigo’: ca. 5.0 mg/g DW (whole fruit)	Mal-glc-fer-rut<br>Mal-glc-coum-rut<br>Del-glc-coum-rut<br>Del-glc-caf-rut<br>Pet-glc-fer-rut<br>Pet-glc-coum-rut	[213]
CHI/*AmDel*/*AmRos1*:<br>(*CHI* × *AmDel*/*AmRos1 lines*)	cv. 'Rubion'	*CHI*/*Del*/*Ros1*: 3.25 mg/g FW (peel)<br>*Del*/*Ros1*: 0.8 mg/g FW (peel)<br>WT: 0.008 mg/g FW (peel)<br>*CHI*/*Del*/*Ros1*: ca. 0.31 mg/g FW (flesh)<br>*Del*/*Ros1*: ca.0.01 mg/g FW (flesh)<br>WT: ca. 0.001 mg/g FW (flesh)	NA	[214]
proSlE8:SlAN2-like^{InR}	cv. 'Ailsa Criag'	up to 2.22 mg /g FW (flesh)	NA	[152]
‘Purple’ (*AmDel*/*AmRos1*)	cv. 'Micro-Tom'	14.7 mg/ g DW (whole fruit)	NA	[215]
‘Pink’ (*AmDel*/*AmRos1; f3′f′h*)	'Micro-Tom-like'	1 mg/ g DW (whole fruit)	Cyanindin, Peonidin	[215]
‘Crimson’ (*AmDel*/*AmRos1; AmDFR; f3′5′h*)	'Micro-Tom-like'	5.3 mg/ g DW (whole fruit)	Pel 3-(coum)-rut-5-glc (pelanin)<br>Cya 3-(caf)-rut-5-glc<br>Pel 3-(caf)-rut-5-glc<br>Peo 3-(caf)-rut-5-glc<br>Cya 3-(coum)-rut-5-glc<br>Peo 3-(fer)-rut-5-glc<br>Pel 3-(fer)-rut	[215]
‘Magenta’ (*AmDel*/*AmRos1; AmDFR; AtMYB12; f3′5′h*)	cv. 'Micro Tom-like'	7.9 mg/ g DW (whole fruit)	Peo 3-(coum)-rut-5-glc (peonanin)<br>Peo 3-(caf)-rut-5-glc<br>Cya 3-(coum)-rut-5-glc<br>Pel 3-(coum)-rut-5-glc<br>Peo 3-(fer)-rut-5-glc	[215]

‘Indigo’ (*AmDel/AmRos1; AtMYB12*)	cv. 'Micro-Tom'	24.7 mg/ g DW (whole fruit)	Pet 3-(coum)-rut-5-glc (petanin)<br>Del 3-(caf)-rut-5-glc<br>Del 3-(coum)-rut-5-glc<br>Pet 3-(caf)-rut-5-glc<br>Del 3-(fer)-rut-5-glc<br>Pet 3-(fer)-rut-5-glc<br>Mal 3-(coum)-rut-5-glc	[215]
